# High-fructose diet–induced gut dysbiosis as a mechanistic driver of chronic inflammation and accelerated biological aging: a comprehensive review

**DOI:** 10.3389/fmicb.2026.1865281

**Published:** 2026-08-13

**Authors:** Xin-zhu Xu, Mo-si Dong, Jian-yu Dai, Qing-yan Wang, Xuan-chi Dong, Ying Jin

**Affiliations:** Liaoning University of Traditional Chinese Medicine, Shenyang, China

**Keywords:** epigenetic clock, gut dysbiosis, high-fructose diet, immunosenescence, inflammaging, metabolic endotoxemia, NLRP3 inflammasome, senescence-associated secretory phenotype

## Abstract

High-fructose diet (HFD) has emerged as a critical dietary risk factor for metabolic syndrome, non-alcoholic fatty liver disease (NAFLD), and cardiovascular disease. This review delineates the molecular mechanisms underlying the pathological cascade linking high-fructose intake, gut dysbiosis, chronic inflammation, and accelerated aging. At the metabolic level, excess fructose is rapidly phosphorylated by ketohexokinase (KHK), depleting intracellular ATP while driving overproduction of uric acid and reactive oxygen species (ROS). These events activate the NLRP3 inflammasome and NF-κB signaling, promoting hepatic *de novo* lipogenesis. At the microbial level, chronic fructose loading selectively depletes butyrate-producing taxa (*Faecalibacterium prausnitzii*, *Roseburia* spp.) while enriching Gram-negative Proteobacteria pathobionts, elevating luminal lipopolysaccharide (LPS) concentrations. Butyrate deficiency downregulates tight junction proteins (ZO-1, occludin, claudin-1), compromising intestinal barrier integrity, enabling systemic LPS translocation and establishing metabolic endotoxemia. At the inflammatory level, circulating LPS sustains TLR4–MyD88–NF-κB axis activation, driving chronic overexpression of TNF-α, IL-6, and IL-1β, establishing a self-reinforcing “inflammaging” network centered on the dysbiosis → leaky gut → endotoxemia → NF-κB → NLRP3 loop. At the aging level, sustained inflammatory signaling accelerates biological aging through five interconnected pathways: (1) cellular senescence and paracrine propagation of the senescence-associated secretory phenotype (SASP); (2) ROS-mediated telomeric oxidative damage coupled with DNA damage response–NF-κB activation; (3) mitochondrial dysfunction driving SASP transcription via the mtROS–cGAS–STING axis; (4) inflammation-induced epigenetic clock acceleration through aberrant DNMT3A/B targeting and asymmetric H3K27ac/me3 remodeling; and (5) immunosenescence encompassing TEMRA accumulation, Treg dysfunction, and M1 macrophage polarization, closing a self-sustaining inflammatory loop. At the intervention level, five strategic categories are synthesized: dietary modification (fructose restriction, Mediterranean diet); probiotic and prebiotic microbial restoration; bile acid FXR/TGR5 modulation with exogenous SCFA supplementation; senolytic, NAD⁺ precursor, and mTOR/AMPK-targeted pharmacotherapy; and fecal microbiota transplantation for systemic reconstitution. Clinical evidence demonstrates measurable improvements in glycemic regulation, insulin sensitivity, and SASP attenuation, though individual responses are substantially modulated by baseline microbiota composition. This review provides an integrative framework for understanding HFD-driven accelerated aging and establishes an evidence base for multilevel, precision-guided anti-inflammaging interventions.

## 1 Introduction

The widespread incorporation of high-fructose corn syrup (HFCS) into sugar-sweetened beverages and ultra-processed foods has driven a sustained increase in per capita dietary fructose intake over recent decades (Jung et al., 2022). Excessive fructose consumption is strongly associated with the development of obesity, type 2 diabetes mellitus (T2DM), non-alcoholic fatty liver disease (NAFLD), and cardiovascular disease (Jung et al., 2022). Unlike glucose, fructose is preferentially catabolized via the fructolysis pathway in the small intestine and liver, bypassing key rate-limiting checkpoints of glycolysis; this leads to the overproduction of lipogenic substrates and uric acid, accelerating metabolic stress (Herman and Birnbaum, 2021). When fructose intake exceeds absorptive capacity, unabsorbed fructose enters the distal intestine, where it serves as a fermentable substrate that disrupts microbial composition and impairs intestinal barrier function, promoting elevated circulating endotoxin levels (Garcia et al., 2022). The gut-mediated mechanisms by which dietary fructose induces systemic metabolic injury remain incompletely characterized (Sindhunata et al., 2022).

The gut microbiome maintains host metabolic homeostasis through its preservation of epithelial barrier integrity, modulation of immune responses, and production of bioactive metabolites including short-chain fatty acids (SCFAs), secondary bile acids, and indole-derived compounds (Khaledi et al., 2024). High-fructose feeding rapidly induces dysbiosis, characterized by selective depletion of beneficial taxa—including *Lactobacillus*, *Bifidobacterium*, and butyrate-producing Firmicutes—and concomitant expansion of Gram-negative pathobionts (Jung et al., 2022). Butyrate, the principal energy substrate of colonocytes, exerts anti-inflammatory and epigenetic regulatory effects through inhibition of histone deacetylases (HDACs) and activation of the G protein-coupled receptors GPR41 and GPR109a (Salvi and Cowles, 2021). Depletion of butyrate-producing communities reduces expression of tight junction proteins—including claudin-1, occludin, and ZO-1—resulting in increased paracellular intestinal permeability (Salvi and Cowles, 2021). Expansion of Gram-negative bacteria elevates luminal lipopolysaccharide (LPS) concentrations; subsequent LPS translocation across the compromised epithelial barrier into the systemic circulation establishes metabolic endotoxemia, persistently activating the TLR4–NF-κB signaling axis and sustaining chronic systemic inflammation (Teodori et al., 2019).

Inflammaging—the chronic, sterile, low-grade inflammatory state that develops progressively with advancing age—is mechanistically defined by persistent activation of nuclear factor-κB (NF-κB) and sustained overexpression of pro-inflammatory cytokines, including TNF-α, IL-6, and IL-1β (Teodori et al., 2019). This pathological state constitutes a shared mechanistic substrate underlying multiple age-related chronic diseases, including obesity, T2DM, cardiovascular disease, and neurodegenerative disorders (Di Giosia et al., 2022). Gut dysbiosis is a critical upstream driver of inflammaging: the resultant increase in intestinal permeability and enhanced systemic LPS translocation directly engage innate immune pathways, initiating and sustaining the inflammatory cascade (Caetano-Silva et al., 2024). Age-related enhancement of gut microbiota TLR4 immunogenicity drives persistent systemic endotoxemia and mucosal inflammatory infiltration, providing direct experimental evidence linking microbial aging signatures to heightened inflammatory tone (Caetano-Silva et al., 2024). Sustained NF-κB activation and oxidative stress induce cellular senescence, triggering the senescence-associated secretory phenotype (SASP), which amplifies paracrine inflammation and accelerates telomere attrition (López-Otín et al., 2023). Immunosenescence and inflammaging are mutually reinforcing: functionally exhausted immune cells lose the capacity to resolve inflammatory signals, sustaining a self-amplifying cycle of systemic inflammation (Santoro et al., 2021). Chronic inflammation has been formally established as a core hallmark of biological aging, operating in concert with genomic instability, mitochondrial dysfunction, and cellular senescence within an integrated aging framework (López-Otín et al., 2023).

Mechanistic reviews that integrate the complete cascade from high-fructose intake through gut dysbiosis, metabolite perturbation, and chronic inflammation to accelerated biological aging remain limited (Ren et al., 2023). Existing literature predominantly addresses isolated mechanistic nodes, without providing an integrative, cross-level interpretation of this sequential cascade. This review addresses four principal scientific questions: (1) How does a high-fructose diet specifically remodel the gut microbiome to selectively drive pathobiont expansion? (2) How does dysbiosis disrupt key metabolic axes, including SCFAs, secondary bile acids, tryptophan metabolism, and trimethylamine N-oxide (TMAO)? (3) How do metabolite imbalances establish and sustain chronic systemic inflammation through the LPS–TLR4–NF-κB axis and NLRP3 inflammasome activation? (4) How does chronic inflammation accelerate biological aging through cellular senescence, epigenetic clock advancement, and immunosenescence? This review aims to construct an integrative mechanistic framework linking high-fructose dietary patterns to accelerated aging via the gut microbiome–inflammation axis, providing an evidence-based rationale for nutritional interventions and microbiome-targeted therapeutic strategies.

## 2 Effects of a high-fructose diet on the gut microecology

### 2.1 Intestinal metabolic pathways and absorption mechanisms of fructose

The consumption of high-fructose corn syrup (HFCS) in sugar-sweetened beverages and processed foods has increased substantially over recent decades, and excessive fructose intake is closely associated with the rising prevalence of metabolic syndrome, non-alcoholic fatty liver disease (NAFLD), and intestinal inflammation (Herman and Birnbaum, 2021).

Fructose is absorbed predominantly at the apical membrane of small intestinal epithelial cells (enterocytes) via facilitated diffusion mediated by glucose transporter 5 (GLUT5, SLC2A5), the only member of the GLUT family with absolute substrate specificity for fructose, operating independently of Na⁺ gradients or ATP (Koepsell, 2020). After entering the enterocyte cytosol, fructose is exported into the portal vein via basolateral GLUT2 (SLC2A2), which additionally transports glucose and galactose, though with lower affinity for fructose (Ferraris et al., 2018).

Upon entry into enterocytes, fructose is rapidly phosphorylated by ketohexokinase (KHK) to form fructose-1-phosphate (F1P). Unlike hexokinase, KHK lacks allosteric feedback inhibition, resulting in rapid depletion of intracellular ATP (Herman and Birnbaum, 2021). Under hypoxic conditions at the villus tip, F1P binds and inhibits the M2 isoform of pyruvate kinase (PKM2), stabilizing its catalytically less-active form and indirectly supporting hypoxia-inducible factor-1α (HIF-1α) signaling. In murine models fed high-fructose corn syrup for 4 weeks, this mechanism prolongs enterocyte survival at the villus tip and produces a 25–40% increase in villus length specifically in the duodenum and proximal jejunum, thereby expanding the absorptive surface area (Taylor et al., 2021).

The small intestinal capacity for fructose transport is finite. When fructose intake exceeds this capacity, unabsorbed fructose spills over into the distal ileum and colon, where it serves as a highly fermentable substrate for the colonic microbiota, initiating a cascade of systemic perturbations to the intestinal microecology (Herman and Birnbaum, 2021).

### 2.2 Effects of high fructose intake on gut microbiota diversity

Alpha diversity indices (e.g., Shannon index, species richness) reflect within-sample biodiversity, while beta diversity metrics (e.g., principal coordinate analysis, PCoA) capture inter-group community structural differences. Both are central indicators of microecological health, and a high-fructose diet exerts meaningful effects on each.

In animal models, mice fed a high-fructose diet demonstrated significantly reduced gut microbiota alpha diversity—as measured by the Shannon index and total operational taxonomic unit (OTU) count—compared with normal diet controls (*p* < 0.01) (Taylor et al., 2021). Mice administered HFCS for 16 weeks exhibited marked separation in beta diversity (PCoA) from controls, with substantial compositional changes at the genus level (Shen et al., 2022).

In human studies, a crossover dietary intervention enrolling 12 healthy women—sequentially assigned to low-fructose (<10 g/d), fruit-rich high-fructose (100 g/d), and HFCS-supplemented high-fructose (100 g/d) phases—revealed that the HFCS phase produced a distinct microbiota compositional profile compared with the fruit-rich high-fructose phase, characterized by a reduction in Firmicutes abundance, an increase in Bacteroidetes, and a decrease in butyrate-producing genera such as *Faecalibacterium* (Beisner et al., 2020). These contrasting effects underscore the critical role of the fructose source and co-ingested dietary matrix—particularly fiber content—in determining microbial outcomes.

At the phylum level, the Firmicutes/Bacteroidetes (F/B) ratio is a widely used marker of gut dysbiosis; its increase is associated with obesity, while a decrease is linked to inflammatory bowel disease (Magne et al., 2020). High-fructose and high-sugar diets broadly shift the F/B ratio, and this compositional disruption is closely associated with host insulin resistance and systemic low-grade inflammation (Magne et al., 2020).

### 2.3 Selective expansion of pathogenic taxa: genus- and species-level changes

Beyond the generalized reduction in microbiota diversity, a high-fructose diet exerts selective substrate pressure that favors the expansion of specific opportunistic and potentially pathogenic taxa.

### 2.4 Proteobacteria expansion and metabolic endotoxemia

Mice fed a high-fructose diet for 12 weeks exhibited a significant increase in the relative abundance of Proteobacteria, accompanied by elevated serum lipopolysaccharide (LPS) levels, increased intestinal permeability, and impaired glucose tolerance (Do et al., 2018). As gram-negative bacteria, Proteobacteria members—particularly the genus *Escherichia*—are rich in cell wall LPS. Their expansion increases luminal LPS concentrations, and upon translocation across a compromised intestinal barrier, circulating LPS activates hepatic TLR4–NF-κB signaling, triggering metabolic endotoxemia and systemic low-grade inflammation (Guney et al., 2023).

### 2.5 Expansion of *Bacteroides* and *Veillonella*

High-fructose feeding significantly promotes the expansion of *Bacteroides* species, whose overgrowth stimulates the proliferation of intraepithelial lymphocytes (IELs) in the intestinal epithelium and drives upregulation of pro-inflammatory cytokines IL-1β and IL-6 (Taylor et al., 2021) The genus *Veillonella* is also enriched following HFCS dietary intervention; certain *Veillonella* species can ferment fructose and incorporate it into their LPS structure, potentially amplifying intestinal endotoxin production (Beisner et al., 2020).

### 2.6 Enrichment of additional pro-inflammatory taxa

16S rRNA gene sequencing of HFCS-fed mice identified 39 differentially abundant genera, of which 13 were enriched, including *Parasutterella*, *Mucispirillum*, and *Helicobacter*—taxa with established pro-inflammatory properties (Han et al., 2022). The enrichment of these genera has been repeatedly validated in murine models of inflammatory bowel disease, suggesting that high-fructose diet-driven microbiota remodeling may create a permissive environment for intestinal inflammatory disease phenotypes.

### 2.7 Suppression of beneficial microbiota and the formation of dysbiosis

Concurrent with the expansion of opportunistic taxa, a high-fructose diet exerts a profound suppressive effect on multiple classes of health-promoting commensal bacteria, constituting the opposing pole of a bidirectional dysbiotic shift.

### 2.8 Depletion of short-chain fatty acid-producing bacteria

Short-chain fatty acids (SCFAs) are the primary end-products of colonic microbial fermentation of dietary fiber; discrete fiber structures selectively promote the expansion of specific SCFA-producing taxa and thereby precisely regulate the production ratios of butyrate, propionate, and acetate (Deehan et al., 2020). Following HFCS intervention, the abundance of key butyrate-producing genera—including *Faecalibacterium*—was significantly reduced, and a negative correlation was observed between the total Firmicutes abundance and plasma LDL cholesterol levels (Beisner et al., 2020). The suppression of SCFA-producing microbiota and the consequent reduction in fecal SCFA concentrations have been confirmed across multiple animal and human studies (Jung et al., 2022).

In a murine model of non-alcoholic steatohepatitis (NASH) induced by a high-fructose, high-fat diet, the relative abundance of *Faecalibacterium prausnitzii* was markedly depleted. Supplementation with this bacterium alleviated hepatic injury and restored microbiota composition, providing direct evidence of its protective role against high-fructose diet-related metabolic damage (Shin et al., 2023).

### 2.9 Alterations in gut microbiota composition and intestinal barrier function

In rats fed a high-fructose diet, the F/B ratio was elevated, and intestinal tissue levels of pro-inflammatory cytokines (IL-1β, TNF-α) were increased. Concurrently, the expression of tight junction proteins including occludin and claudin-1 was reduced, indicating impaired intestinal barrier integrity (Yalçın Buğdaycı et al., 2024). Importantly, this fructose-driven barrier impairment is amenable to dietary intervention: supplementation with the dietary fiber inulin—by remodeling the small intestinal microbiome with *Bacteroides acidifaciens* as a key mediator—promotes the catabolism of dietary fructose prior to its colonic spillover, thereby reversing fructose-induced hepatic steatosis (Jung et al., 2025).

### 2.10 Depletion of *Akkermansia muciniphila*

*Akkermansia muciniphila*, a member of the phylum Verrucomicrobia that colonizes the intestinal mucus layer, maintains gut barrier function through mucus layer renewal and regulation of the Th17/Treg immune balance; its depletion is associated with increased risk of metabolic syndrome, obesity, and type 2 diabetes mellitus (Rodrigues et al., 2022). *A. muciniphila* reinforces the expression of tight junction proteins in intestinal epithelial cells through the secretion of its outer membrane protein Amuc_1100 and other effector molecules, thereby preserving intestinal permeability homeostasis (Mo et al., 2024).

### 2.11 Integrated mechanisms underlying dysbiosis formation

Fructose overflow into the colon confers a substrate-based competitive advantage to opportunistic pathobionts, displacing fiber-dependent SCFA-producing commensals from their ecological niches (Herman and Birnbaum, 2021). The resultant SCFA depletion, combined with reduced *A. muciniphila* abundance, impairs intestinal barrier function. Subsequent LPS translocation via the gut–liver axis activates TLR4 signaling, establishing a self-reinforcing cycle of “dysbiosis → leaky gut → inflammation” (Guney et al., 2023). This microecological pathological process represents a critical mechanistic intermediary linking high-fructose diet exposure to the pathogenesis of metabolic disease (Liu et al., 2020).

**Figure 1:**
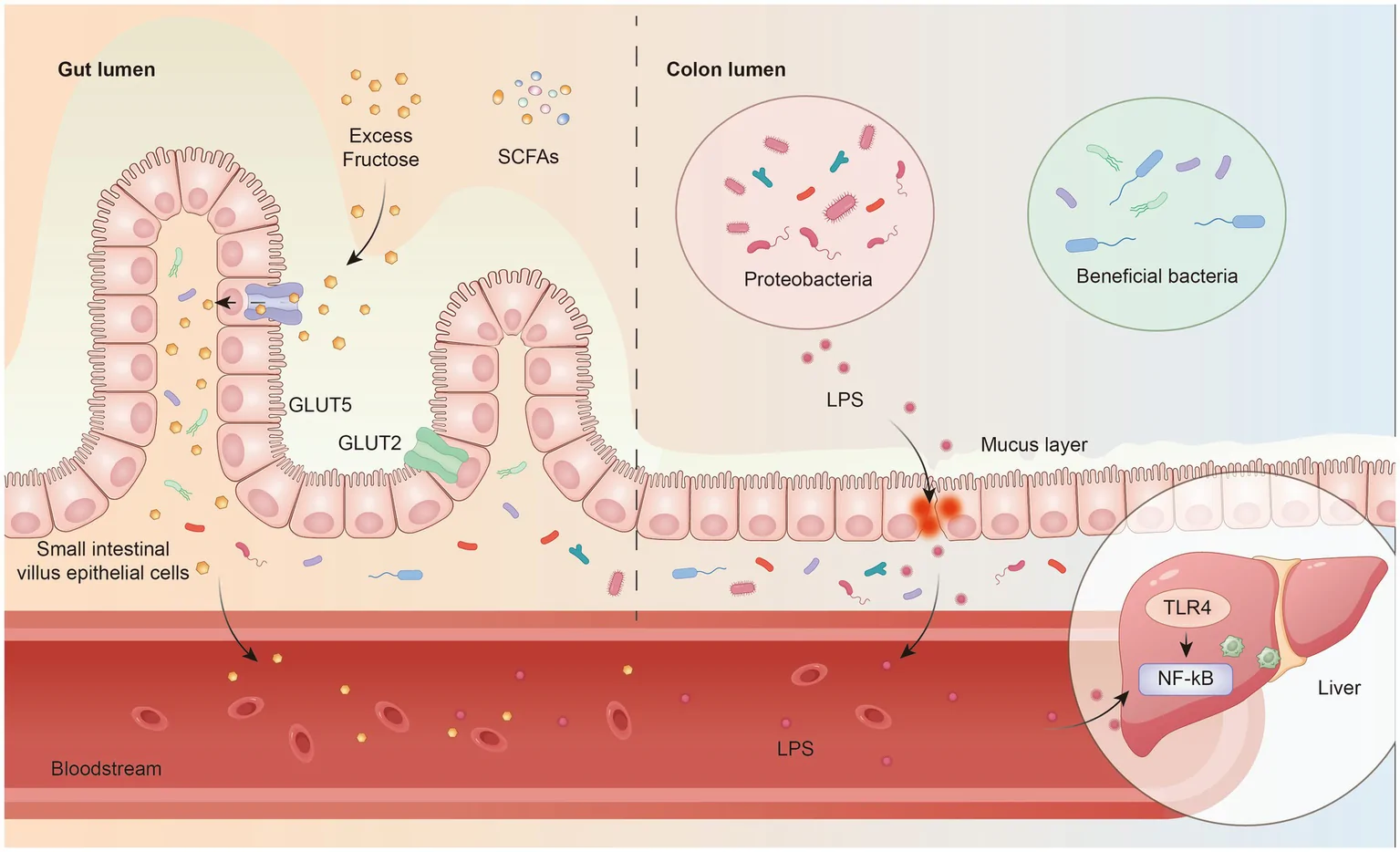
High-fructose diet–induced perturbation of intestinal microecology and initiation of the gut–liver inflammatory axis. Dietary fructose is absorbed at the apical membrane of small intestinal enterocytes via GLUT5 (SLC2A5) and exported to the portal circulation through basolateral GLUT2 (SLC2A2). When intake exceeds the finite small intestinal absorptive capacity, unabsorbed fructose spills into the distal ileum and colon, where it serves as a fermentable substrate that selectively reshapes the colonic microbiota. The resulting dysbiosis is characterized by expansion of Gram-negative pathobionts (predominantly Proteobacteria) and depletion of beneficial commensals, including butyrate-producing Firmicutes and *Akkermansia muciniphila*, with consequent reduction of luminal short-chain fatty acids (SCFAs). SCFA depletion impairs colonocyte energy metabolism and downregulates tight junction proteins (ZO-1, occludin, claudin-1), while mucus layer thinning further compromises barrier integrity. The resulting “leaky gut” enables paracellular translocation of lipopolysaccharide (LPS) into the portal and systemic circulation, establishing metabolic endotoxemia. Portal LPS activates the TLR4–NF-κB axis in hepatic Kupffer cells, initiating chronic low-grade inflammation along the gut–liver axis. GLUT, glucose transporter; LPS, lipopolysaccharide; NF-κB, nuclear factor κB; SCFAs, short-chain fatty acids; TLR4, Toll-like receptor 4; ZO-1, zonula occludens-1.

The integrated mechanism by which a high-fructose diet remodels the intestinal microecology and initiates gut-derived systemic inflammation is summarized in Figure 1. Excess dietary fructose that escapes GLUT5/GLUT2-mediated small intestinal absorption spills over into the colon, where it drives selective expansion of Gram-negative pathobionts and depletion of SCFA-producing commensals; the resultant SCFA deficiency and mucus layer thinning compromise epithelial barrier integrity, enabling LPS translocation into the systemic circulation and activating hepatic TLR4–NF-κB signaling.

## 3 Alterations in metabolite profiles driven by gut microbiota dysbiosis

Gut microbiota dysbiosis is not merely a structural shift in microbial composition but is more profoundly manifested as a comprehensive remodeling of the host metabolite landscape. Through fermentation, biotransformation, and biosynthesis, gut microorganisms continuously produce key small-molecule metabolites that regulate host physiological homeostasis. Upon the onset of dysbiosis, the yield, diversity, and proportional balance of these metabolites undergo changes that mediate intestinal barrier disruption, immune activation, and metabolic dysfunction.

### 3.1 Reduction of short-chain fatty acids and intestinal barrier injury

Short-chain fatty acids (SCFAs), primarily acetate (C2), propionate (C3), and butyrate (C4), are the principal metabolic products generated by colonic microbial fermentation of dietary fiber, with total luminal concentrations typically reported in the range of 50–150 mmol/L in healthy adults, as originally established by direct post-mortem measurements across the human large bowel (Hays et al., 2024; Cummings et al., 1987). Butyrate is predominantly produced by *Faecalibacterium prausnitzii*, *Roseburia intestinalis*, and *Eubacterium hallii* within the Firmicutes phylum; propionate is mainly derived from Bacteroidetes and *Akkermansia muciniphila*; while acetate is broadly synthesized by Bacteroidetes and bifidobacteria (Martin-Gallausiaux et al., 2021).

SCFAs exert multidimensional physiological functions. With respect to energy supply, butyrate serves as the preferred respiratory fuel of colonocytes, accounting for approximately 70–80% of their energetic demands via mitochondrial β-oxidation and the tricarboxylic acid (TCA) cycle, as originally demonstrated in isolated human and rat colonocyte preparations (Salvi and Cowles, 2021; Roediger, 1980). Regarding barrier function, SCFAs upregulate the expression of tight junction proteins (ZO-1, claudin-1, and occludin), thereby reducing intestinal permeability partly through stabilization of hypoxia-inducible factor-1α (HIF-1α) (Liu et al., 2018). In terms of signaling, SCFAs function as endogenous ligands for G protein-coupled receptors GPR41 (FFA3), GPR43 (FFA2), and GPR109A (HCA2), regulating enteroendocrine function (GLP-1 and PYY secretion) and immune cell activation (Lee et al., 2024). From an epigenetic standpoint, butyrate acts as a histone deacetylase (HDAC) inhibitor, upregulating genes encoding tight junction proteins and anti-inflammatory mediators while suppressing NF-κB activation and reducing production of pro-inflammatory cytokines such as TNF-α and IL-6 (Martin-Gallausiaux et al., 2021).

During dysbiosis, the abundance of SCFA-producing bacteria is broadly diminished, and reduced butyrate concentration constitutes a shared metabolic signature of inflammatory bowel disease (IBD), metabolic syndrome, and colorectal cancer (Deleu et al., 2021). Butyrate deficiency impairs β-oxidation in colonocytes, elevates intraluminal oxygen tension, and fosters the outgrowth of aerobic Proteobacteria, further perpetuating dysbiosis (Hays et al., 2024). Attenuation of HDAC inhibition results in NF-κB pathway activation, compromising the integrity of the intestinal epithelial barrier (Martin-Gallausiaux et al., 2021). Different dietary fiber substrates selectively regulate the butyrate-to-propionate ratio, thereby precisely modulating SCFA composition (Deehan et al., 2020). High-fructose diets suppress SCFA-producing commensal bacteria and reduce the diversity of fermentable substrates, significantly decreasing total fecal SCFA content (Tan et al., 2021). Propionate enters the portal circulation and participates in hepatic gluconeogenesis and lipid metabolism regulation, while acetate contributes to lipid synthesis and immune modulation in the systemic circulation. Any form of dysbiosis that globally diminishes SCFA production compromises host intestinal homeostasis from both metabolic and barrier perspectives (Portincasa et al., 2022).

### 3.2 Elevated LPS and endotoxin levels: metabolic endotoxemia

Lipopolysaccharide (LPS), a core structural component of the outer membrane of Gram-negative bacteria, is composed of lipid A, a core oligosaccharide, and an O-antigen polysaccharide chain, with lipid A constituting the principal determinant of endotoxin activity (Raetz and Whitfield, 2002). The interaction between LPS and the TLR4 signaling pathway underlies its pathological effects: LPS binds serum LPS-binding protein (LBP) and is presented to CD14, which transfers it to the MD-2/TLR4 heterodimer, thereby activating intracellular MyD88-dependent and TRIF-dependent signaling cascades, leading to nuclear translocation of NF-κB and IRF3 and triggering massive production of pro-inflammatory mediators including TNF-α, IL-1β, IL-6, and type I interferons (Pålsson-McDermott and O'Neill, 2004).

Under physiological conditions, the intestinal epithelial barrier effectively prevents LPS from entering the systemic circulation. During dysbiosis, disruption of the intestinal barrier allows LPS to enter the portal vein and systemic circulation via the paracellular route or through transcellular transport associated with chylomicrons, causing a chronic low-grade elevation of serum LPS levels—a condition termed “metabolic endotoxemia” (Fuke et al., 2019). This condition was originally defined as a sustained, diet-induced 2- to 3-fold elevation of plasma LPS concentration above fasted baseline, which drives chronic systemic low-grade inflammation through persistent TLR4 activation, thereby promoting the development of insulin resistance, nonalcoholic fatty liver disease (NAFLD), and atherosclerosis (Cani et al., 2007; Violi et al., 2023).

High-fat, high-sugar diets promote metabolic endotoxemia through multiple mechanisms: (1) increasing the relative abundance of Proteobacteria (LPS-producing bacteria); (2) depleting SCFA-producing commensals and impairing the intestinal epithelial barrier; and (3) enabling LPS to bind chylomicrons upon co-ingestion with dietary fat, thereby bypassing hepatic detoxification and entering the peripheral circulation (Cani et al., 2007). LPS activates hepatic Kupffer cells via TLR4 through the gut–liver axis, inducing NF-κB-mediated inflammatory cascades that initiate hepatic lipid metabolic disturbances and fibrogenesis (An et al., 2022). Sustained endotoxin stimulation impairs insulin signaling in skeletal muscle and adipose tissue by promoting activation of JNK and IKKβ pathways and inducing serine phosphorylation of insulin receptor substrate-1 (IRS-1), representing a key pathological basis for obesity-associated type 2 diabetes mellitus (Tanti and Jager, 2009).

LPS derived from *Escherichia coli* is highly acylated and potently pro-inflammatory, whereas LPS from *Bacteroides* spp. is hypoacylated and exhibits substantially weaker TLR4-activating capacity (Vatanen et al., 2016). The increased ratio of Proteobacteria to Bacteroidetes during dysbiosis further amplifies the inflammatory consequences of metabolic endotoxemia (Guney et al., 2023). Moreover, chronic endotoxemia activates the NLRP3 inflammasome, promoting the maturation of IL-18 and IL-1β and amplifying intestinal inflammation, thereby establishing a self-reinforcing circuit of “endotoxemia–leaky gut–inflammation” (Platnich and Muruve, 2019).

### 3.3 Dysregulation of secondary bile acid metabolism

Bile acids are amphipathic sterol molecules synthesized in the liver from cholesterol as a precursor. Primary bile acids [chenodeoxycholic acid (CDCA) and cholic acid (CA)] are conjugated with glycine or taurine in the liver prior to secretion into the small intestine via bile, where they facilitate lipid emulsification. Gut microorganisms perform two core biotransformations: bile salt hydrolase (BSH)-mediated deconjugation and 7α-dehydroxylation, converting CA to deoxycholic acid (DCA) and CDCA to lithocholic acid (LCA)—the secondary bile acids (Guzior and Quinn, 2021).

Upon activation, the farnesoid X receptor (FXR) induces fibroblast growth factor 19 (FGF19) to suppress hepatic cholesterol 7α-hydroxylase (CYP7A1), providing negative feedback regulation of bile acid synthesis (Chiang and Ferrell, 2022). FXR activation also upregulates the expression of ileal tight junction proteins (occludin and claudin-1), thereby reinforcing the integrity of the intestinal epithelial barrier (Verbeke et al., 2015). Activation of the G protein-coupled bile acid receptor TGR5 promotes GLP-1 secretion from intestinal L cells and inhibits NLRP3 inflammasome phosphorylation and ubiquitination in macrophages, thereby exerting anti-inflammatory effects (Han et al., 2023).

During dysbiosis, the abundance of 7α-dehydroxylating bacteria markedly declines, leading to decreased secondary bile acid production, an elevated ratio of primary bile acids, and impaired physiological activation of FXR and TGR5 (Wahlström et al., 2016). The direct consequences include: (1) attenuated FXR signaling → decreased FGF19 secretion → disinhibition of CYP7A1 → excessive bile acid synthesis; (2) high concentrations of DCA inducing DNA damage and apoptosis in colonic epithelial cells by disrupting mitochondrial membrane potential and promoting reactive oxygen species (ROS) generation; and (3) a shift of the bile acid pool toward a more hydrophobic (cytotoxic) composition (Fiorucci et al., 2018).

The gut microbiota influences host lipid metabolism by altering bile acid pool composition and modulating FXR signaling—a mechanism directly validated in germ-free mouse models (Parséus et al., 2017). Secondary bile acids promote intestinal stem cell (ISC) proliferation via the TGR5–SRC–YAP signaling axis to maintain epithelial integrity; when secondary bile acids are depleted, the regenerative capacity of the intestinal barrier is diminished (Sorrentino et al., 2020). A bidirectional interaction exists between bile acids and the microbiota: bile acids regulate intestinal microbial colonization through direct antimicrobial effects and FXR-induced antimicrobial peptide production, while the microbiota composition reciprocally determines bile acid pool composition (Molinero et al., 2019).

### 3.4 Shifts in tryptophan metabolic pathways: indole pathway vs. kynurenine pathway

Tryptophan (Trp) is an essential amino acid with diverse metabolic fates: approximately 95% is metabolized via the kynurenine (Kyn) pathway, approximately 5% is converted via the serotonin (5-HT) pathway, and the remaining fraction is directly metabolized by gut microorganisms to yield indole and its derivatives (Xue et al., 2023).

Indole pathway: Gut bacteria harboring tryptophanase activity—including *Lactobacillus* spp., *Clostridium sporogenes*, and *Bacteroides* spp.—catabolize tryptophan to indole, which is further metabolized to derivatives such as indole-3-propionic acid (IPA), indole-3-acetic acid (IAA), and indole-3-aldehyde (IAld) (Agus et al., 2018). These indole derivatives serve as endogenous ligands for the aryl hydrocarbon receptor (AhR); AhR activation induces intestinal epithelial IL-22 production, upregulates tight junction proteins and MUC2 expression, promotes Treg differentiation, and maintains intestinal immune homeostasis (Xiao et al., 2021). Indole can also directly stimulate GLP-1 secretion from intestinal L cells, regulating gastrointestinal motility and insulin secretion (Hyland et al., 2022). IPA, by virtue of its antioxidant properties and activation of the pregnane X receptor (PXR), further reinforces tight junction protein expression and constitutes a critical indole derivative for intestinal barrier protection (Agus et al., 2021).

Kynurenine pathway: IDO1 and IDO2 in intestinal epithelial cells and immune cells, together with hepatic TDO, convert tryptophan to kynurenine (Kyn) (Munn and Mellor, 2016). IDO1 is potently induced by pro-inflammatory cytokines including IFN-γ and TNF-α. Kyn serves as the precursor to multiple downstream metabolites, ultimately generating NAD⁺ via quinolinate phosphoribosyltransferase; certain branch pathways produce neurotoxic metabolites such as quinolinic acid (QA) (Cervenka et al., 2017).

During dysbiosis, tryptophan metabolic pathways undergo significant deviation. Depletion of indole-producing bacteria leads to reduced AhR activation and diminished IL-22 production, impairing intestinal epithelial barrier repair, reducing GLP-1 secretion, and slowing colonic motility (Gao et al., 2020). Chronic inflammation drives excessive IDO1 activation, diverting tryptophan flux toward the kynurenine pathway and reducing the supply of 5-HT precursors, resulting in intestinal 5-HT deficiency and attenuated colonic peristaltic reflexes (Yano et al., 2015). Accumulation of downstream metabolites such as QA exerts neurotoxic effects, contributing to the pathogenesis of depression, anxiety, and cognitive impairment through the gut–brain axis (Cervenka et al., 2017). Kynurenine activates FoxP3 transcription via the IDO–Kyn–AhR axis, driving T-cell differentiation toward a regulatory T cell (Treg) phenotype, thereby creating a tumor immunosuppressive microenvironment that promotes colorectal cancer development and progression (Zhang et al., 2021).

The tryptophan metabolic shift during dysbiosis is characterized by a bidirectional pattern—attenuated indole pathway and overactivated kynurenine pathway: reduced production of beneficial indole metabolites diminishes the protective function of the AhR–IL-22 axis, while inflammation-driven IDO1 activation promotes kynurenine accumulation, depleting tryptophan reserves and causing both 5-HT deficiency and neurotoxic metabolite accumulation (Ye et al., 2022). This metabolic remodeling integrates intestinal microecological dysregulation with enteric nervous system dysfunction, neuropsychiatric disorders, and colorectal tumorigenesis within a unified pathological framework.

### 3.5 Changes in other key metabolites (TMAO, succinate, and others)

**Figure 2:**
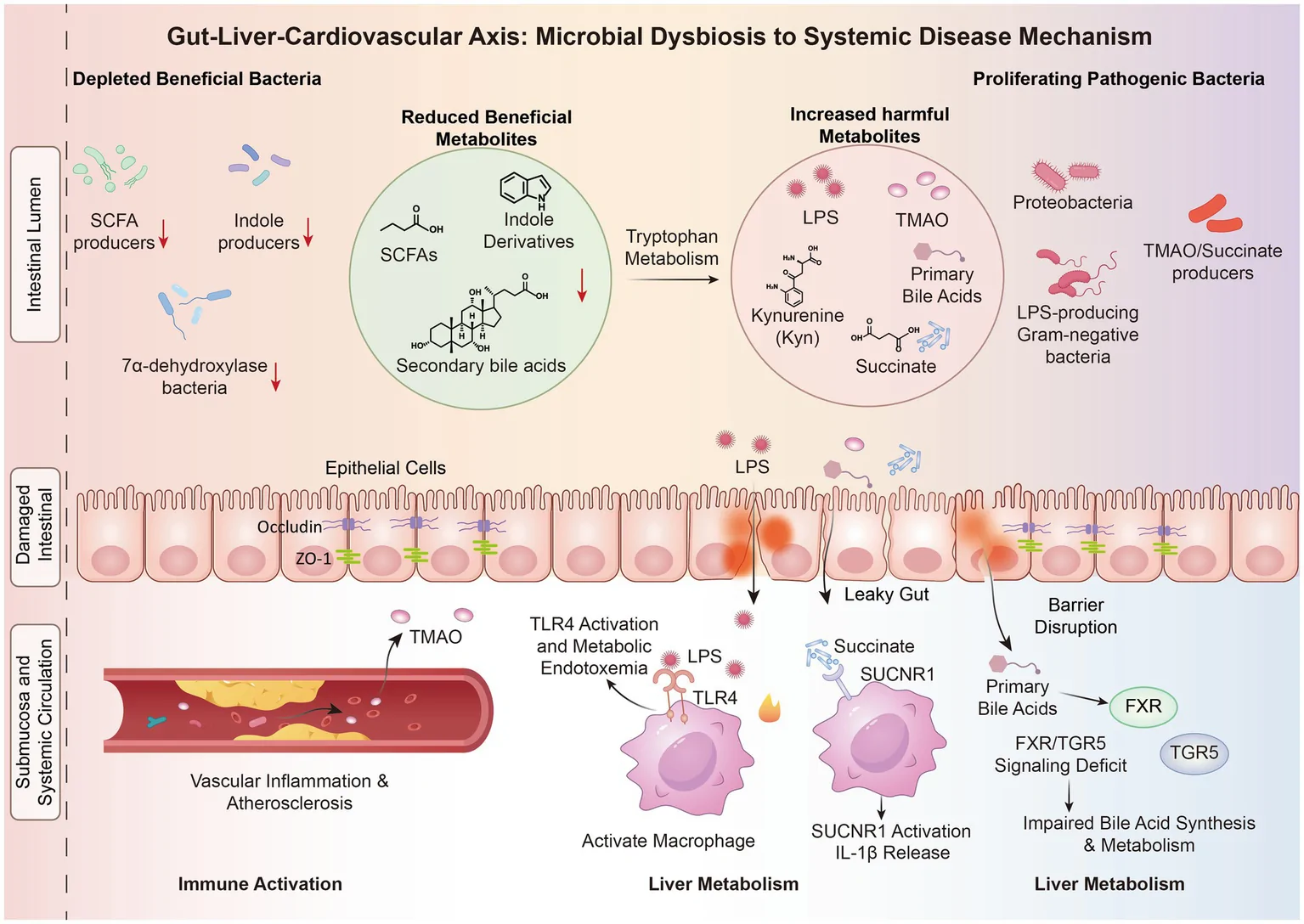
Gut microbial dysbiosis remodels the host metabolite landscape and drives the gut–liver–cardiovascular axis of systemic disease. Dysbiosis induces a bidirectional taxonomic shift, depleting beneficial taxa (SCFA-, indole-, and secondary bile acid–producing bacteria) while expanding pathobionts (Proteobacteria; *Prevotella*-driven TMAO/succinate producers). The luminal metabolite pool is correspondingly remodeled: beneficial mediators—SCFAs, indole derivatives (IPA, IAA, IAld), and secondary bile acids (DCA, LCA)—are reduced, while harmful metabolites (LPS, TMAO precursors, kynurenine, primary bile acids, succinate) accumulate; tryptophan flux diverts from the indole pathway toward the kynurenine pathway. SCFA depletion downregulates tight junction proteins, generating “leaky gut” and enabling translocation of LPS, primary bile acids, and other metabolites into the lamina propria and portal circulation. Three parallel cascades ensue: (i) LPS–TLR4–NF-κB activation drives metabolic endotoxemia and macrophage cytokine release; (ii) succinate–SUCNR1 signaling stabilizes HIF-1α, priming NLRP3-mediated IL-1β maturation; (iii) elevated primary-to-secondary bile acid ratio impairs FXR/TGR5 signaling. In parallel, hepatic FMO3-derived TMAO drives vascular endothelial inflammation and atherogenesis. DCA, deoxycholic acid; FMO3, flavin-containing monooxygenase 3; FXR, farnesoid X receptor; HIF-1α, hypoxia-inducible factor-1α; IAA/IAld/IPA, indole-3-acetic acid/aldehyde/propionic acid; Kyn, kynurenine; LCA, lithocholic acid; LPS, lipopolysaccharide; NF-κB, nuclear factor κB; NLRP3, NOD-like receptor pyrin domain–containing 3; SCFAs, short-chain fatty acids; SUCNR1, succinate receptor 1; TGR5, Takeda G protein-coupled receptor 5; TLR4, Toll-like receptor 4; TMAO, trimethylamine N-oxide.

The systemic remodeling of the host metabolite landscape driven by gut microbial dysbiosis, together with its downstream effects on the intestinal barrier, hepatic metabolism, and cardiovascular system, is summarized in Figure 2. The depletion of beneficial metabolites (SCFAs, indole derivatives, secondary bile acids) and the parallel accumulation of harmful metabolites (LPS, TMAO, kynurenine, succinate, primary bile acids) jointly establish a multi-axis pathological cascade encompassing immune activation, hepatic metabolic dysregulation, and vascular inflammation.

### 3.6 Trimethylamine N-oxide

Trimethylamine N-oxide (TMAO) is generated through a gut microbiota–liver metabolic axis: dietary phosphatidylcholine, L-carnitine, and related precursors are cleaved by gut microorganisms harboring CutC lyase or carnitine oxidase activity—including *Prevotella*, *Clostridium*, and *Fusobacterium*—to produce trimethylamine (TMA), which is subsequently oxidized by hepatic flavin-containing monooxygenase 3 (FMO3) to yield TMAO (Zhen et al., 2023).

TMAO promotes atherosclerosis through multiple mechanisms: activation of MAPK and NF-κB signaling to drive vascular inflammation, inhibition of reverse cholesterol transport to promote foam cell formation, and activation of the NLRP3 inflammasome to facilitate IL-1β maturation (Seldin et al., 2016). Plasma TMAO levels in patients with heart failure are independently associated with all-cause mortality and adverse cardiovascular event risk (Tang et al., 2014). High-fat, high-sugar diet-driven microbial compositional shifts—characterized by enrichment of TMAO-producing bacteria such as *Prevotella*—augment TMA production; since FMO3 activity is regulated by bile acid signaling, dysbiosis further amplifies TMAO generation by modulating FMO3 expression (Chen et al., 2016). TMAO is also associated with chronic intestinal inflammation: elevated TMAO can activate NF-κB signaling in intestinal epithelial cells to induce inflammatory responses, while dysbiosis in IBD patients frequently alters the composition of TMAO-metabolizing microorganisms (Liu et al., 2020).

### 3.7 Succinate

Succinate is a central intermediate of the TCA cycle and an important fermentation product of gut microorganisms including *Prevotella*, *Bacteroides*, and *Phascolarctobacterium*, with luminal concentrations normally maintained at low levels in the healthy colon (Dunn et al., 2016). During dysbiosis, exuberant expansion of *Prevotella* leads to marked succinate accumulation in the colonic lumen—a phenomenon confirmed in the fecal metabolome of pediatric IBD patients (Dunn et al., 2016) and recapitulated in metabolomic analyses of adult IBD cohorts (Fernández-Veledo and Vendrell, 2019).

Succinate initiates an immune-activating cascade by binding its receptor SUCNR1 (GPR91): SUCNR1 activation promotes dendritic cell maturation, enhances pro-inflammatory cytokine secretion (including IL-1β), and drives Th17 cell polarization (Ferraris et al., 2018). Intracellularly accumulated succinate inhibits prolyl hydroxylase domain (PHD) enzymes, thereby stabilizing HIF-1α, upregulating IL-1β transcription, and promoting the pro-inflammatory M1 macrophage phenotype, thus sustaining a persistent inflammatory microenvironment (Tannahill et al., 2013). Elevated *Prevotella copri*-derived succinate is associated with disease activity in rheumatoid arthritis and other systemic autoimmune conditions; overproduction of succinate under dysbiotic conditions can directly exacerbate joint inflammation, indicating that succinate serves as a critical metabolic signal linking gut microbiota dysbiosis to extra-intestinal immune pathology (Jiang et al., 2022).

### 3.8 Other key metabolites

Hydrogen sulfide (H₂S): Produced by sulfate-reducing bacteria such as *Desulfovibrio* spp. At low concentrations, H₂S exerts mucosal-protective effects; however, at high concentrations—markedly elevated during dysbiosis—H₂S inhibits mitochondrial cytochrome c oxidase in colonic epithelial cells, causing mucosal injury and disrupting tight junctions (Blachier et al., 2019). Branched-chain fatty acids (BCFAs): Isobutyrate, isovalerate, and related compounds are generated from protein fermentation; their relative increase during dysbiosis serves as a metabolic marker of excessive microbial protein fermentation and insufficient fiber fermentation (Neis et al., 2015). Uremic toxin precursors: *Clostridium* and related bacteria metabolize tryptophan and tyrosine to produce indole and p-cresol, which are absorbed by the host and sulfated in the liver to form indoxyl sulfate (IS) and p-cresyl sulfate (pCS); these compounds accumulate in the context of renal impairment, exacerbating systemic inflammation and oxidative stress (Castillo-Rodriguez et al., 2018). γ-Aminobutyric acid (GABA): Produced by *Lactobacillus* and *Bifidobacterium*, reduced GABA production during dysbiosis may impair enteric sensory nerve function and contribute to visceral hypersensitivity (Strandwitz et al., 2019). Polyamines: Spermidine, spermine, and related compounds synthesized by *Bacteroides* and *Fusobacterium* promote intestinal epithelial cell proliferation; aberrant polyamine metabolism under dysbiotic conditions is associated with increased colorectal cancer risk (Shen et al., 2020).

In summary, dysbiosis-driven remodeling of the host metabolite landscape is both comprehensive and systemic: SCFA depletion impairs barrier function; LPS release drives metabolic endotoxemia; disruption of secondary bile acid metabolism disables the FXR/TGR5 signaling axis; tryptophan metabolic deviation toward the kynurenine pathway precipitates neuroimmune dysregulation; and elevated TMAO and succinate further amplify cardiovascular and systemic inflammatory risk. These metabolic alterations are mutually reinforcing and collectively constitute the core pathological metabolic network through which gut microbiota dysbiosis drives metabolic disease.

## 4 Metabolite-mediated mechanisms of chronic inflammation activation

**Figure 3:**
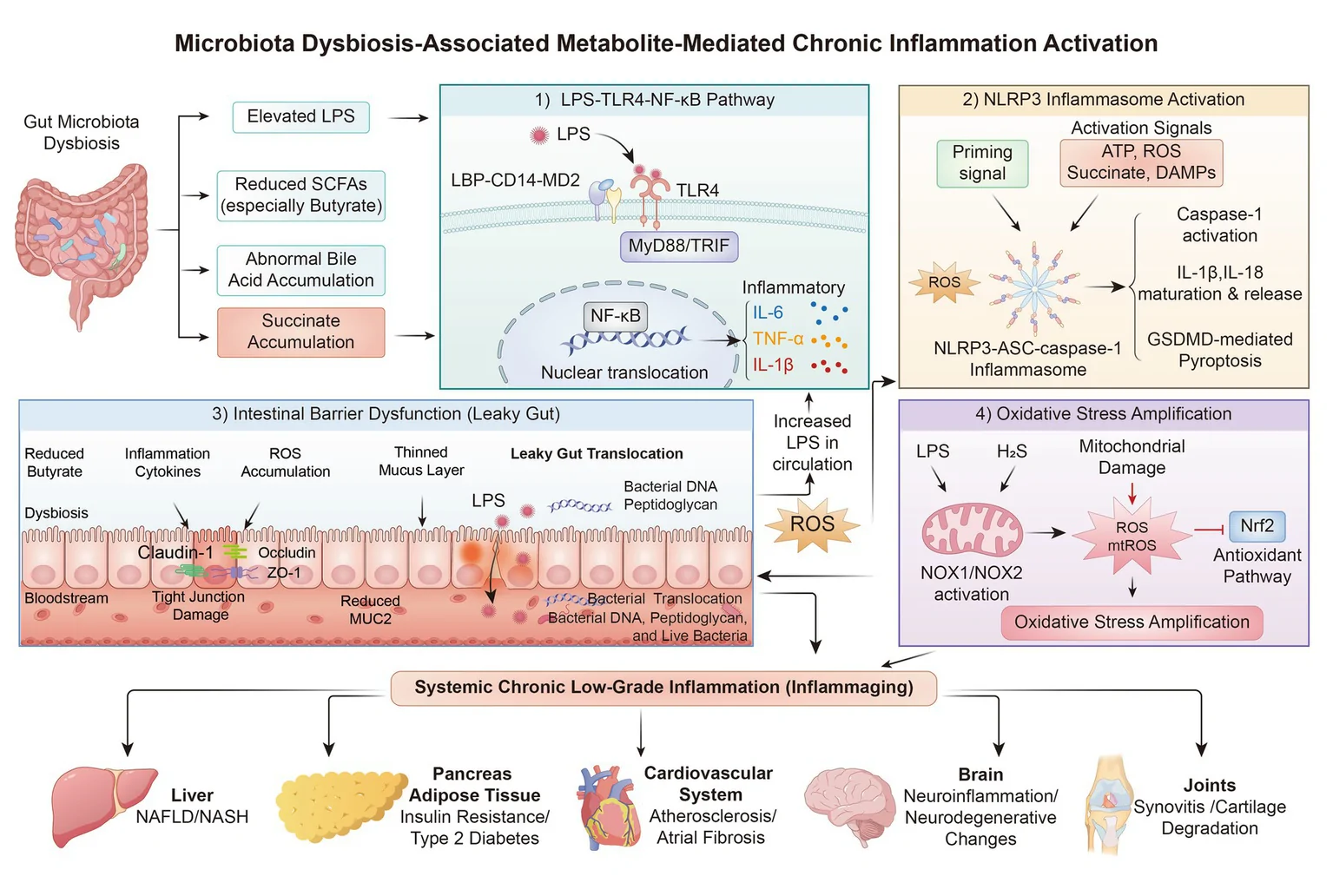
Metabolite-mediated four-tiered amplification network of chronic inflammation driven by gut microbial dysbiosis. Four upstream metabolic signals—elevated LPS, reduced SCFAs (particularly butyrate), aberrant bile acid accumulation, and succinate accumulation—converge to drive a self-reinforcing four-tiered inflammatory network. (1) LPS–TLR4–NF-κB pathway (primary amplifier): LPS, captured by the LBP–CD14–MD2 complex, activates TLR4-mediated MyD88- and TRIF-dependent cascades, driving NF-κB nuclear translocation and transcription of IL-6, TNF-α, and IL-1β. (2) NLRP3 inflammasome activation (secondary amplifier): TLR4–NF-κB priming, together with activation signals (ATP, mtROS, succinate, DAMPs), assembles the NLRP3–ASC–caspase-1 complex; activated caspase-1 matures IL-1β/IL-18 and cleaves gasdermin D to execute pyroptosis. (3) Intestinal barrier dysfunction (self-renewing signal source): Butyrate deficiency, cytokine accumulation, ROS overload, and mucinolytic dysbiosis disrupt tight junctions and thin the mucus layer, perpetuating LPS translocation. (4) Oxidative stress amplification (cross-activating mediator): NOX1/NOX2 activation and impaired Nrf2 antioxidant defense elevate ROS/mtROS, which oxidize tight junction proteins, re-activate TLR4 via lipid peroxidation products, and promote NLRP3 oligomerization. Their convergence establishes non-resolving “inflammaging,” driving NAFLD/NASH, type 2 diabetes, atherosclerosis, neurodegeneration, and synovitis. ASC, apoptosis-associated speck-like protein; CD14, cluster of differentiation 14; DAMPs, damage-associated molecular patterns; IL, interleukin; LBP, LPS-binding protein; LPS, lipopolysaccharide; MD2, myeloid differentiation factor 2; mtROS, mitochondrial ROS; MyD88, myeloid differentiation primary response 88; NAFLD/NASH, non-alcoholic fatty liver disease/steatohepatitis; NF-κB, nuclear factor κB; NLRP3, NOD-like receptor pyrin domain–containing 3; NOX, NADPH oxidase; Nrf2, nuclear factor erythroid 2-related factor 2; ROS, reactive oxygen species; SCFAs, short-chain fatty acids; TLR4, Toll-like receptor 4; TNF-α, tumor necrosis factor α; TRIF, TIR-domain-containing adaptor-inducing interferon-β.

The remodeling of the metabolite landscape triggered by gut dysbiosis converts local intestinal microecological imbalance into persistent systemic chronic inflammation through cascade-amplified molecular signaling. Metabolites including lipopolysaccharide (LPS), diminished short-chain fatty acids (SCFAs), aberrant bile acids, and succinate collectively constitute upstream drivers of inflammatory signaling, while downstream mechanisms encompass sustained activation of pattern recognition receptors, inflammasome assembly with effector cytokine release, disruption of the intestinal physical barrier, and systemic propagation of oxidative stress. These mechanisms are mutually nested and self-reinforcing, ultimately establishing a chronic low-grade inflammatory homeostasis that cannot be spontaneously resolved—termed “inflammaging”—which serves as the common pathological backdrop for a broad spectrum of metabolic, cardiovascular, and neurodegenerative diseases. The four-tiered, mutually nested mechanism by which dysbiosis-driven metabolite remodeling is converted into systemic chronic inflammation is summarized in Figure 3. The TLR4/NF-κB pathway functions as the primary amplifier, the NLRP3 inflammasome as the secondary amplifier, intestinal barrier disruption as a self-renewing signal source, and oxidative stress as a cross-activating mediator; the long-term convergence of these four tiers ultimately establishes the chronic low-grade inflammatory homeostasis termed inflammaging, which drives pathological progression across multiple organ systems (Table 1).

**Table 1:** Mapping of intervention strategies to their target node on the high-fructose–microbiota–inflammation–aging axis and current evidence tier.

Strategy	Primary target node on the axis	Proposed mechanism/effect	Current evidence tier (this review)
Fructose/added-sugar restriction; MedDiet; high-fiber; intermittent fasting	Stage 1—upstream KHK/uric-acid/ROS input	Lowers KHK-driven metabolic injury; raises microbial diversity and SCFA output; reinforces the barrier	Human RCTs + reviews (Wang et al., 2024; Yang J. et al., 2024; Lim and Nam, 2023; Zhao et al., 2023; Zhu et al., 2024; Mishra et al., 2024)
Probiotics/prebiotics/synbiotics	Stage 2—microbial ecology and SCFA capacity	Restores beneficial taxa; augments SCFA; strain- and timing-dependent; baseline-microbiota-dependent	RCT (incl. *n* = 200) + reviews (Roshanravan et al., 2018; Hodgkinson et al., 2023; Hickson et al., 2019; Islam et al., 2023)
Bile-acid (FXR/TGR5) modulation; exogenous SCFA/butyrate	Downstream—barrier and inflammatory effector nodes	FXR/TGR5 signaling suppresses NLRP3/NF-κB; butyrate (HDACi, GPR41/43) restores tight junctions	Animal models + small RCTs (Wahlström et al., 2016; Guarente et al., 2024; Qiu et al., 2023; Zhang et al., 2024; Horvath et al., 2024; Zhang et al., 2023)
Senolytics, senomorphics, NAD⁺ precursors, metformin, rapamycin	Stage 4—senescent cells/SASP/NAD⁺/mTOR	Clears senescent cells or suppresses SASP; replenishes NAD⁺; modulates AMPK/mTOR	Phase 1/early human + preclinical (Hickson et al., 2019; Islam et al., 2023; Guarente et al., 2024)
Fecal microbiota transplantation (FMT)	Stage 2—systems-level microbiota reconstruction	Whole-community transfer; short-term glycemic/insulin benefit; response is baseline-dependent	Meta-analysis (9 RCTs) + Phase 2 (Qiu et al., 2023; Zhang et al., 2024; Horvath et al., 2024)

KHK, ketohexokinase; ROS, reactive oxygen species; SCFA, short-chain fatty acid; MedDiet, Mediterranean diet; FXR, farnesoid X receptor; TGR5, Takeda G protein-coupled receptor 5; HDACi, histone deacetylase inhibitor; SASP, senescence-associated secretory phenotype; FMT, fecal microbiota transplantation.

### 4.1 Sustained activation of the TLR4/NF-κB signaling pathway

Toll-like receptor 4 (TLR4) is the core pattern recognition receptor of the innate immune system for detecting LPS and is broadly expressed on intestinal epithelial cells, macrophages, dendritic cells, and vascular endothelial cells. During gut dysbiosis, increased intestinal barrier permeability enables LPS to continuously translocate across the epithelium into the portal vein and systemic circulation, triggering chronic low-grade TLR4 signaling activation (Violi et al., 2023).

Following LPS–LBP complex formation, LPS is presented to CD14 and subsequently transferred to the MD-2/TLR4 heterodimer (Di Vincenzo et al., 2024). TLR4 activation simultaneously initiates two downstream signaling cascades: the MyD88–TIRAP-dependent pathway is triggered at the plasma membrane, activating IRAK1/4 and TRAF6, leading to IKK complex phosphorylation, IκB degradation, and nuclear translocation of NF-κB (p65/p50 heterodimer), which drives transcription of pro-inflammatory genes including TNF-α, IL-1β, and IL-6; the TRAM–TRIF-dependent pathway is activated in endosomes following TLR4 internalization, inducing type I interferon production via IRF3 nuclear translocation (Di Vincenzo et al., 2024; Ciesielska et al., 2021). In the context of metabolic endotoxemia, sustained exposure to low-concentration LPS maintains moderate NF-κB activation, driving basal secretion of pro-inflammatory cytokines and establishing a “gut leakage–endotoxemia” positive feedback circuit (Violi et al., 2023; Yang J. et al., 2024).

The TLR4-activating potency of LPS differs markedly by bacterial origin: highly acylated *E. coli* LPS functions as a potent agonist, whereas hypo-acylated LPS produced by *Bacteroides* species acts as a TLR4 antagonist (Vatanen et al., 2016). During dysbiosis, the relative expansion of Proteobacteria at the expense of Bacteroidetes shifts circulating LPS toward a highly pro-inflammatory species composition, thereby exacerbating dysregulated TLR4/NF-κB activation (Ciesielska et al., 2021).

Sustained NF-κB activation results in multi-organ injury. In the liver, TLR4–NF-κB signaling in Kupffer cells drives TNF-α and IL-6 secretion, activates hepatic stellate cells, and promotes progression from non-alcoholic fatty liver disease (NAFLD) to non-alcoholic steatohepatitis (NASH) (Yang M. et al., 2024). NF-κB also transactivates the adhesion molecules VCAM-1, ICAM-1, and MCP-1, promoting atherosclerosis; trimethylamine N-oxide (TMAO) further amplifies vascular endothelial inflammatory responses through the MAPK–NF-κB axis (Seldin et al., 2016). The pivotal role of NF-κB at the intersection of autoimmunity and inflammation renders it a central node through which gut dysbiosis drives systemic chronic inflammation (Barnabei et al., 2021).

SCFA deficiency and NF-κB hyperactivation act synergistically: under physiological conditions, butyrate maintains controlled histone deacetylation of NF-κB target gene promoters through histone deacetylase (HDAC) inhibition; upon butyrate depletion, this epigenetic constraint is released, chromatin accessibility at NF-κB target loci increases, and the inflammatory transcriptional program becomes sustained (Deleu et al., 2021).

### 4.2 NLRP3 inflammasome assembly and release of IL-1β and IL-18

Building upon the priming signal provided by TLR4/NF-κB, diverse metabolites generated during gut dysbiosis further activate the NLRP3 inflammasome, triggering the maturation and secretion of IL-1β and IL-18—constituting the second key step in metabolite-mediated inflammatory amplification (Fu and Wu, 2023).

The NLRP3 inflammasome is a tripartite complex comprising the sensor protein NLRP3, the adaptor protein ASC, and the effector protein pro-caspase-1. Its activation requires two sequential signals: the first signal, provided by TLR4–NF-κB, transcriptionally upregulates NLRP3 and pro-IL-1β; the second signal is delivered by danger signals including extracellular ATP, monosodium urate crystals, cholesterol crystals, mitochondrial reactive oxygen species (mtROS), and lysosomal damage (Fu and Wu, 2023; Zheng et al., 2020). In the context of gut dysbiosis, LPS provides the priming signal, while gut metabolites (saturated fatty acids, excess succinate) and circulating damage-associated molecular patterns (DAMPs) that enter the bloodstream following gut barrier disruption constitute the activation signals (Yang et al., 2023).

Upon NLRP3 activation, ASC is recruited to form a macromolecular speck complex that assembles and activates pro-caspase-1. Activated caspase-1 proteolytically cleaves pro-IL-1β and pro-IL-18 to yield their biologically active forms; it simultaneously cleaves gasdermin D (GSDMD), whose N-terminal domain inserts into the plasma membrane to form large pores, triggering pyroptosis (Mangan et al., 2018). Pyroptosis, accompanied by rapid osmotic cell lysis, leads to large-scale extracellular release of IL-1α, high-mobility group box 1 (HMGB1), ATP, and other danger-associated mediators, which further activate NLRP3 in neighboring cells, establishing an inflammatory amplification positive feedback loop (Mangan et al., 2018).

At the level of specific metabolites, LPS and ROS generated by aberrant glucose metabolism act synergistically in the NLRP3 activation mechanism underlying dysbiosis-driven cardiovascular complications, including atrial fibrillation (Zhang et al., 2022). Succinate accumulation stabilizes HIF-1α by inhibiting prolyl hydroxylases (PHDs), and HIF-1α directly drives transcription of NLRP3 target genes, forming a “metabolic–inflammasome” axis (Tannahill et al., 2013). Elevated succinate derived from *Prevotella copri* participates in driving rheumatoid arthritis inflammation by promoting IL-1β release downstream of NLRP3 activation (Jiang et al., 2022). When SCFAs are deficient, the negative regulation of NLRP3 by butyrate—mediated through GPR109A signaling and HDAC inhibition—is attenuated, indirectly facilitating inflammasome activation (Deleu et al., 2021).

IL-1β exerts pathological effects in multiple target organs: in pancreatic β-cells, sustained IL-1β signaling impairs insulin secretory function; in the myocardium, IL-1β drives the NLRP3–atrial fibrosis cascade; in articular synovium, IL-1β activates matrix metalloproteinase (MMP)-mediated cartilage degradation (Jiang et al., 2022; Fu and Wu, 2023). IL-18 drives IFN-γ production by NK cells and T cells, contributing to the perpetuation of chronic inflammation in autoimmune diseases and inflammatory bowel disease (IBD) (Fu and Wu, 2023). In IBD, NLRP3 activation and gut dysbiosis form a bidirectional reinforcing loop: dysbiosis activates NLRP3, promoting IL-1β and IL-18 secretion, which further disrupts the intestinal barrier and precipitates more severe dysbiosis (Direito et al., 2023). In hyperuricemia, gut dysbiosis promotes tissue injury through activation of the renal NLRP3 inflammasome, indicating that this mechanism exerts cross-organ systemic effects (Zhou et al., 2024).

### 4.3 Increased intestinal barrier permeability and systemic inflammation induced by “leaky gut”

The integrity of the intestinal epithelial barrier depends on precise regulation of the tight junction (TJ) protein complex, consisting primarily of transmembrane proteins (claudins, occludin, JAM-A) and cytoplasmic scaffold proteins (ZO-1, ZO-2, ZO-3) (Chae et al., 2024; Macura et al., 2024). During gut dysbiosis, metabolite-induced barrier damage leads to “leaky gut,” triggering systemic inflammatory responses (Chae et al., 2024).

The molecular mechanisms by which gut dysbiosis damages the barrier operate at multiple levels. Butyrate deficiency deprives colonocytes of their primary energy substrate, impairs HIF-1α stabilization, and downregulates claudin-1 and occludin expression (Liu et al., 2018). TNF-α and IL-1β activate myosin light-chain kinase (MLCK), triggering actomyosin ring contraction and epithelial gap widening; TNF-α additionally induces occludin internalization via a caveolin-1-dependent mechanism, directly disrupting TJ architecture (Salvi and Cowles, 2021). Excess ROS directly oxidizes key cysteine residues within TJ proteins, impairing their interaction with ZO-1 and causing TJ assembly defects (Dmytriv et al., 2024).

Disruption of the mucus barrier is equally consequential. During gut dysbiosis, mucinolytic pathogenic microorganisms directly degrade MUC2 polymers; concurrently, SCFA depletion—particularly butyrate deficiency—undermines the nutritional support that butyrate normally provides to colonocyte differentiation and goblet cell MUC2 secretion, resulting in mucus layer thinning and elimination of the protective distance between microorganisms and epithelial cells (Hays et al., 2024).

The “lumen-to-blood translocation” resulting from gut barrier disruption constitutes an upstream trigger of systemic inflammation (Macura et al., 2024). Translocated contents include LPS or LPS–chylomicron complexes (which enter the lymphatic system via lacteals, bypassing hepatic first-pass metabolism to reach the peripheral circulation directly), bacterial peptidoglycan fragments (activating NOD1/NOD2), bacterial DNA (activating TLR9), and intact viable bacteria (Cani et al., 2007). Under high-fat diet conditions, the LPS–chylomicron translocation route is particularly critical, enabling LPS to directly induce inflammation in adipose tissue, vascular walls, and myocardium (Cani et al., 2007).

Following transplantation of gut microbiota from obese donor mice into normoglycemic recipients, the recipients exhibited increased intestinal permeability, augmented systemic inflammation, and impaired glucose tolerance, directly establishing the causal chain of dysbiosis → intestinal leakage → metabolic inflammation (Mishra et al., 2023). Serum zonulin levels are significantly elevated in patients with type 2 diabetes mellitus and positively correlate with HOMA-IR and circulating pro-inflammatory cytokine concentrations (Macura et al., 2024).

Leaky gut and gut dysbiosis constitute a self-perpetuating bidirectional circuit: dysbiosis → barrier damage → LPS in bloodstream → NF-κB activation → TNF-α/IL-1β release → MLCK activation → TJ protein disruption → more severe barrier damage → further LPS translocation → exacerbated dysbiosis (Fuke et al., 2019; Chae et al., 2024). Increased intestinal permeability also activates lamina propria dendritic cells and macrophages, amplifying the systemic propagation of inflammatory signals through Th17/Treg imbalance (Di Vincenzo et al., 2024).

### 4.4 Amplification of oxidative stress and reactive oxygen species accumulation

In the context of gut dysbiosis, concurrent increases in ROS production and impairment of ROS clearance disrupt the redox balance, generating oxidative stress that serves as a critical accelerator of chronic inflammation (Mostafavi Abdolmaleky and Zhou, 2024).

The mechanisms by which gut dysbiosis drives excessive ROS accumulation are multifaceted. High concentrations of hydrogen sulfide (H₂S), produced by sulfate-reducing bacteria including *Desulfovibrio* species, directly inhibit mitochondrial electron transport chain complex IV, causing electron leakage and superoxide anion generation, with substantial increases in mtROS (Sun et al., 2024). LPS-induced TLR4/NF-κB activation upregulates NOX1 and NOX2 expression, catalyzing sustained superoxide anion production in macrophages and epithelial cells (Singh et al., 2022). Butyrate deficiency attenuates SCFA-mediated activation of the Nrf2–Keap1 signaling axis: under physiological conditions, butyrate promotes Nrf2 nuclear translocation through HDAC inhibition and FFAR receptor–AMPK signaling, driving expression of antioxidant enzymes including SOD, CAT, GPx, and HO-1; during dysbiosis, reduced butyrate impairs Nrf2 nuclear translocation, diminishing antioxidant defense capacity (Martin-Gallausiaux et al., 2021; Kunst et al., 2023). Pathogenic Enterobacteriaceae generate H₂O₂ under hypoxic conditions, further transferring the oxidative stress burden to host cells (Mostafavi Abdolmaleky and Zhou, 2024).

ROS accumulation induces multi-tiered cellular damage that perpetuates inflammation. ROS-mediated oxidation of polyunsaturated fatty acids generates 4-hydroxynonenal (4-HNE) and malondialdehyde (MDA), which activate TLR4 and NF-κB, establishing a self-amplifying loop of “ROS → lipid peroxidation → TLR4 → NF-κB → more ROS” (Sun et al., 2024). ROS directly oxidize guanine bases in DNA (generating 8-oxo-2′-deoxyguanosine; 8-OHdG), activating ATM/ATR–p53 stress responses and contributing to progressive DNA damage accumulation in colonic epithelium (Li et al., 2023). mtROS induces NLRP3 oligomerization; oxidized mitochondrial DNA directly binds NLRP3 and promotes its conformational activation, connecting oxidative stress and inflammasome activation in a positive feedback loop (Platnich and Muruve, 2019; Mangan et al., 2018).

TMAO activates ROS-dependent NF-κB pathways, promotes superoxide anion production in vascular endothelium, inhibits endothelial nitric oxide synthase (eNOS) activity, and reduces nitric oxide (NO) bioavailability; diminished NO further augments uncoupled eNOS-derived superoxide production, exacerbating endothelial oxidative stress and contributing to atherogenesis (Zhen et al., 2023; Seldin et al., 2016).

*Lactobacillus* and *Bifidobacterium* species secrete antioxidant factors including SOD and catalase, and enhance the antioxidant capacity of intestinal epithelial cells by activating host Nrf2 signaling through SCFAs (Martin-Gallausiaux et al., 2021; Kunst et al., 2023). Gut dysbiosis leads to reduced abundance of these cytoprotective species, creating a compounded insult of “increased pro-oxidative pressure + diminished antioxidant protection” (Mostafavi Abdolmaleky and Zhou, 2024).

### 4.5 Establishment of chronic low-grade inflammation (inflammaging)

The sustained TLR4/NF-κB activation, repeated NLRP3 inflammasome assembly, persistently elevated intestinal barrier permeability, and continuous oxidative stress amplification described above are mutually coupled through co-activation, positive feedback, and signal crosstalk, ultimately establishing a chronic low-grade inflammatory homeostasis that is independent of an identifiable infectious trigger and resistant to physiological anti-inflammatory resolution—termed “inflammaging” (Caldarelli et al., 2024).

The defining features of inflammaging are persistently mildly elevated basal circulating levels of pro-inflammatory cytokines (IL-6, IL-1β, TNF-α), accompanied by progressive deterioration of immune regulatory capacity—including a pro-inflammatory shift in Th17/Treg balance, impaired NK cell function, and the accumulation of terminally differentiated effector memory T and B cells with concomitant depletion of naïve lymphocytes (immunosenescence) (Santoro et al., 2021; Conway and Duggal, 2021). IL-6 is the primary driver of hepatic acute-phase protein (C-reactive protein [CRP], fibrinogen) synthesis; its chronic elevation maintains CRP at mildly abnormal concentrations (1–3 mg/L), constituting an independent predictor of major cardiovascular events (Caldarelli et al., 2024).

Gut dysbiosis participates in the establishment of inflammaging through multiple convergent mechanisms. Sustained LPS-mediated TLR4 activation, succinate-driven NLRP3 priming, and weakened anti-inflammatory barriers due to SCFA depletion collectively maintain low-level NF-κB and NLRP3 activation, driving basal IL-6 and IL-1β secretion (Santoro et al., 2021). ROS accumulation and DNA damage accelerate cellular senescence in epithelial cells and immune cells, inducing expression of the senescence-associated secretory phenotype (SASP)—characterized by abundant secretion of IL-6, IL-8, MMP-3, and other pro-inflammatory mediators—which activates NF-κB in surrounding cells and establishes a “cellular senescence–paracrine inflammation” amplification circuit (Singh et al., 2024). Elderly individuals exhibit diminished gut microbial diversity, reduced abundance of beneficial microorganisms, and expansion of LPS-producing taxa, strengthening LPS-mediated TLR4 inflammatory signaling and accelerating SASP formation and inflammaging progression (Shen et al., 2020; Conway and Duggal, 2021).

Inflammaging accelerates the progression of multiple systemic diseases. IL-1β impairs pancreatic β-cell function, IL-6 drives hepatic insulin resistance, and TNF-α interferes with GLUT4 membrane translocation in adipocytes; these three cytokines synergistically exacerbate type 2 diabetes mellitus progression (Violi et al., 2023; Yang M. et al., 2024). IL-6 and TNF-α can traverse the blood–brain barrier, activate microglia, and induce neuroinflammation, closely implicating this axis in the neurodegenerative changes characteristic of Alzheimer’s disease and Parkinson’s disease (Abdel-Haq et al., 2019). In the cardiovascular system, persistent low-grade inflammation upregulates VCAM-1 and MCP-1, promoting subendothelial monocyte infiltration, foam cell formation, and atherosclerotic plaque destabilization (Seldin et al., 2016).

The bidirectional causal relationship among the microbiome, inflammation, and aging has been validated experimentally. Fecal microbiota transplantation (FMT) from young donor mice into aged recipients significantly reduced circulating inflammatory biomarkers and attenuated aging-associated tissue phenotypes, indicating that the gut microbiota represents a tractable target for inflammaging intervention (Gyriki et al., 2025). Sustained exposure to elevated IL-6 and IL-1β promotes progressive effector memory T-cell accumulation and exhaustion, impairing immune surveillance against infections and malignancies; Treg cells, functionally and numerically suppressed by persistent inflammatory signals, further exacerbate dysregulated pro-inflammatory immune responses (Santoro et al., 2021). This pathological milieu constitutes an important mechanistic basis for the suboptimal antimicrobial and vaccine responses observed in patients with metabolic syndrome.

In summary, the mechanisms by which gut metabolites mediate chronic inflammatory activation constitute a highly integrated pathological network: the TLR4/NF-κB pathway functions as the primary amplifier, the NLRP3 inflammasome as the secondary amplifier, intestinal barrier disruption as a self-renewing signal source, and oxidative stress as a cross-activating mediator; inflammaging represents the irreversible chronic inflammatory homeostasis established through the long-term convergence of these mechanisms. Targeting multiple nodes within this pathological network constitutes a key therapeutic strategy for metabolic diseases and chronic inflammatory conditions.

## 5 Molecular mechanisms by which chronic inflammation accelerates aging

**Figure 4:**
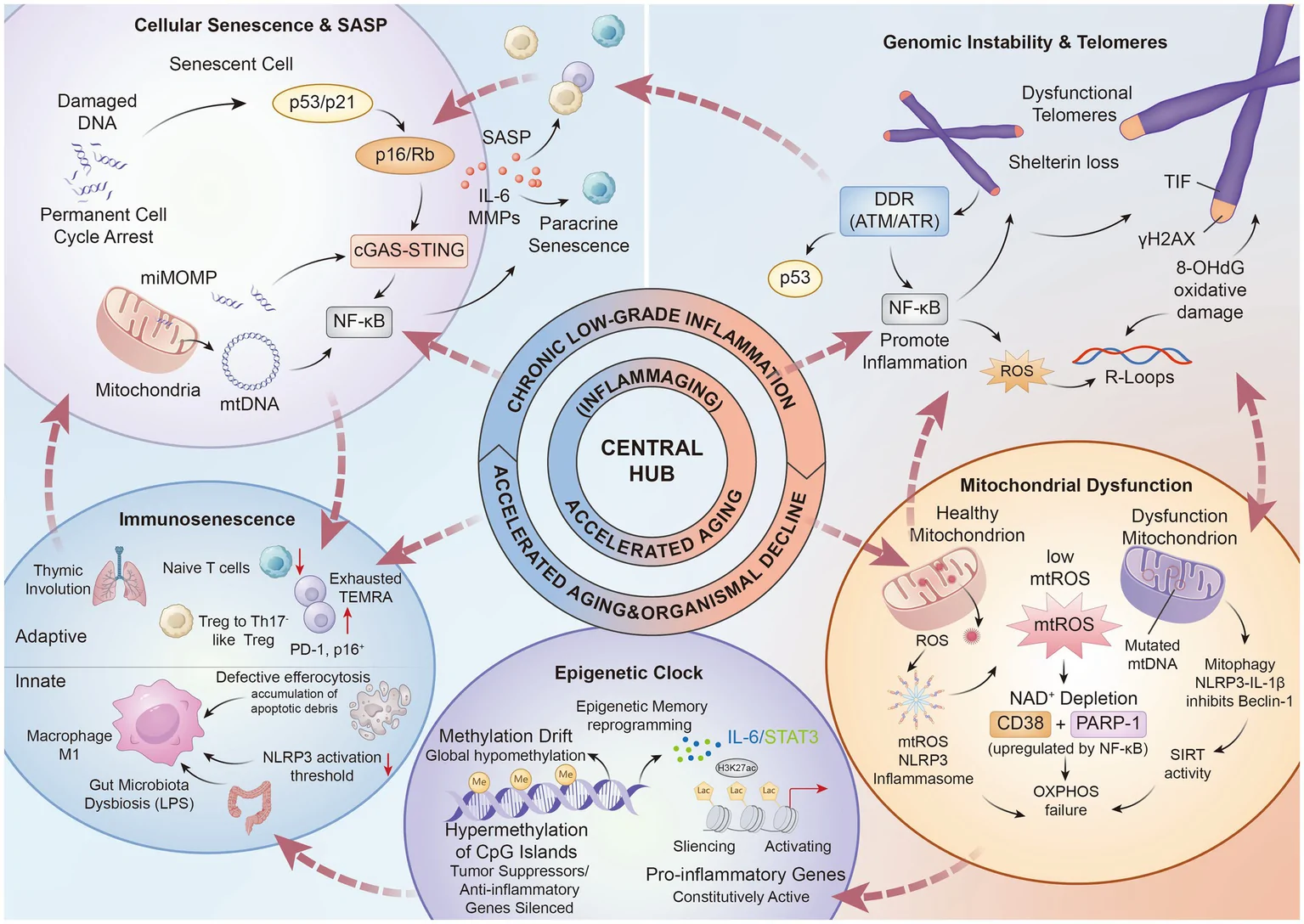
Five interconnected molecular pathways through which inflammaging accelerates biological aging. Inflammaging serves as the central pathological hub orchestrating organismal aging through five mutually reinforcing dimensions, each connected via bidirectional positive feedback. (1) Cellular senescence and SASP: DNA damage drives stable cell cycle arrest via the p53/p21CIP1 and p16INK4a/Rb axes; sublethal mtDNA release activates the cGAS–STING–NF-κB axis, driving SASP transcription that propagates paracrine senescence. (2) Genomic instability and telomere damage: Critically shortened telomeres trigger ATM/ATR-mediated DDR and γH2AX-marked TIFs; ROS-induced 8-OHdG damage at telomeric guanine repeats and replication-stress R-loops further amplify instability, while DDR–NF-κB drives pro-inflammatory transcription. (3) Mitochondrial dysfunction: mtDNA mutations and elevated mtROS accumulate; NLRP3–IL-1β-mediated Beclin-1 inhibition impairs mitophagy, while NF-κB-driven CD38 and PARP-1 upregulation depletes NAD⁺, suppressing SIRT1/SIRT3 activity and culminating in OXPHOS failure. (4) Epigenetic clock acceleration: IL-6/STAT3-driven aberrant DNMT3A/B targeting produces methylation drift; persistent NF-κB activation deposits activating H3K27ac at pro-inflammatory enhancers and repressive H3K27me3 at protective gene promoters, generating “epigenetic memory.” (5) Immunosenescence: Thymic involution and exhausted PD-1⁺/p16INK4a⁺ TEMRA accumulation, IL-6/STAT3-mediated Treg→Th17-like reprogramming, and M1 macrophage polarization with lowered NLRP3 thresholds together lock immune regulation into self-perpetuating dysfunction. 8-OHdG, 8-hydroxy-2′-deoxyguanosine; ATM/ATR, ataxia-telangiectasia mutated/Rad3-related; cGAS, cyclic GMP-AMP synthase; DDR, DNA damage response; DNMT, DNA methyltransferase; γH2AX, phosphorylated H2AX; H3K27ac/me3, H3 lysine 27 acetylation/trimethylation; mtDNA/mtROS, mitochondrial DNA/ROS; NAD⁺, nicotinamide adenine dinucleotide; NF-κB, nuclear factor κB; NLRP3, NOD-like receptor pyrin domain–containing 3; OXPHOS, oxidative phosphorylation; PARP-1, poly(ADP-ribose) polymerase 1; PD-1, programmed cell death protein 1; SASP, senescence-associated secretory phenotype; SIRT, sirtuin; STAT3, signal transducer and activator of transcription 3; STING, stimulator of interferon genes; TEMRA, terminally differentiated effector memory T cells; TIF, telomere dysfunction-induced foci; Treg, regulatory T cell.

Chronic low-grade inflammation (inflammaging) maintains deep bidirectional causal relationships with cellular senescence, genomic instability, mitochondrial dysfunction, epigenetic reprogramming, and immune functional decline, forming a self-reinforcing pathological positive feedback network with the core hallmarks of aging (López-Otín et al., 2023). The following discusses the key mechanisms from five molecular dimensions. The five-dimensional, mutually reinforcing molecular network through which chronic low-grade inflammation accelerates biological aging is summarized in Figure 4. With inflammaging at its central hub, the network encompasses cellular senescence and SASP propagation, telomere damage and genomic instability, mitochondrial dysfunction with NAD⁺ depletion, epigenetic clock acceleration, and immunosenescence; each dimension is connected to the inflammatory core through bidirectional positive feedback, generating a self-amplifying cycle that drives organismal decline.

### 5.1 Cellular senescence and activation of the SASP

Cellular senescence is a state of stable cell cycle arrest driven by diverse stress signals, characterized by irreversible proliferative cessation and multidimensional functional reprogramming, representing a pivotal cellular biological event linking chronic inflammation to organismal aging (Birch and Gil, 2020).

Senescence induction relies on two cooperating pathways. The p53/p21CIP1 pathway is rapidly activated early in the DNA damage response (DDR): ATM/ATR kinases phosphorylate and stabilize p53, driving p21 upregulation that inhibits CDK2 activity and blocks the G₁/S transition (Kumari and Jat, 2021). The p16INK4a/Rb-E2F pathway sustains long-term senescence maintenance: p16INK4a inhibits CDK4/6, maintaining Rb in a persistently hypophosphorylated state that permanently represses transcription of proliferative genes (Kumari and Jat, 2021).

The most pathologically significant feature of senescent cells is the senescence-associated secretory phenotype (SASP). The SASP comprises pro-inflammatory cytokines (IL-6, IL-8, IL-1α), chemokines (CXCL1, CCL2), and matrix metalloproteinases (MMP-3, MMP-9), spreading inflammatory signals to surrounding tissues through paracrine mechanisms; its transcriptional regulation depends on persistent activation of the NF-κB and C/EBPβ transcription factors (Kumari and Jat, 2021).

Mitochondria serve as a critical upstream driver of SASP. In senescent cells, a subset of mitochondria undergo sublethal minority mitochondrial outer membrane permeabilization (miMOMP), releasing mtDNA into the cytosol through BAX/BAK macropores, thereby activating the cGAS-STING pathway and driving large-scale SASP factor transcription through the NF-κB axis (Victorelli et al., 2023).

The SASP induces “paracrine senescence” in neighboring normal cells, causing spatially expansive accumulation of senescent cells within tissues and accelerating overall tissue functional decline (Moiseeva et al., 2023). The cycle of “senescent cell accumulation—SASP secretion—paracrine propagation” constitutes a positive feedback spiral and represents a key amplification node through which chronic inflammation drives systemic aging.

### 5.2 Telomere damage and genomic instability

Telomeres are composed of (TTAGGG)n repeat sequences and rely on the shelterin protein complex (including TRF1, TRF2, and POT1) to maintain their t-loop conformation, preventing chromosome ends from being misrecognized as DNA double-strand breaks (DSBs) (Chakravarti et al., 2021).

With successive cell divisions, telomere sequences progressively shorten due to the end-replication problem and 3′-end processing. When telomeres reach a critical threshold, TRF2 dissociates from the terminus, and exposed telomeric DNA triggers a persistent DDR: ATM phosphorylates H2AX (γH2AX), forming telomere dysfunction-induced foci (TIFs), while simultaneously activating the CHK2-p53-p21 axis to drive cells into irreversible replicative senescence (Rossiello et al., 2022). This DDR signal can also directly induce pro-inflammatory gene transcription through the ATM-NF-κB axis, revealing that telomere damage itself exerts direct pro-inflammatory effects independent of the p53 pathway (Roger et al., 2021).

Telomeres are highly enriched in guanine repeats and are hypersensitive to ROS-induced 8-hydroxyguanine (8-OHdG) modification. Even in the absence of significant telomere shortening, a single induction of 8-OHdG accumulation specifically at telomeric regions is sufficient to trigger hallmarks of p53-dependent senescence, suggesting that elevated ROS in the chronic inflammatory microenvironment can bypass telomere attrition as a rate-limiting step and directly accelerate cellular aging through telomeric oxidative damage (Barnes et al., 2022).

R-loop structures formed during replication stress represent another important source of genomic instability, impeding replisome progression and inducing DSBs; chronic inflammation increases replication stress load through elevated ROS and excessive NF-κB activation, accelerating the accumulation of genomic mutations (López-Otín et al., 2023). The cascade “chronic inflammation → elevated ROS → telomeric oxidative damage/replication stress → DSBs → DDR-NF-κB inflammatory signal reinforcement → further ROS elevation” constitutes one of the core positive feedback mechanisms of self-accelerating aging.

### 5.3 Mitochondrial dysfunction and energy metabolic decline

Mitochondrial dysfunction is one of the most central hallmarks of aging and represents a critical hub for molecular crosstalk between chronic inflammation and cellular senescence (Miwa et al., 2022). Age-associated mitochondrial deterioration involves four intertwined levels: decreased mitochondrial membrane potential (ΔΨm), reduced electron transport chain (ETC) efficiency, excessive mtROS production, and accumulating mtDNA mutations, collectively forming a self-amplifying deterioration cascade. Persistently elevated mtROS drives oxidative mutations in mtDNA; since mtDNA lacks histone protection and has comparatively limited DNA repair mechanisms, its mutation accumulation rate far exceeds that of nuclear DNA, further reducing oxidative phosphorylation (OXPHOS) efficiency and establishing a cascade amplification of “ETC impairment → elevated mtROS → mtDNA mutations → further mitochondrial functional deterioration” (Miwa et al., 2022).

Mitophagy represents the core quality control mechanism for eliminating damaged mitochondria, executed primarily through the PINK1/Parkin pathway. The NLRP3-IL-1β axis activated by chronic inflammation can suppress Beclin-1-mediated autophagy initiation, resulting in accumulation of damaged mitochondria and exacerbating intracellular mtROS burden (Aman et al., 2021). LPS and metabolic toxins derived from gut dysbiosis can directly damage intestinal epithelial and skeletal muscle cell mitochondria, accelerating age-related mitochondrial decline through the gut-mitochondria axis (Picca et al., 2018).

NAD⁺ levels decline significantly with aging, representing an important molecular marker of mitochondrial metabolic deterioration. NAD⁺ is not only a core coenzyme for the ETC but also an essential substrate for deacylases including SIRT1 and SIRT3; its decline weakens SIRT3-mediated deacetylation of mitochondrial proteins, reducing the activity of key antioxidant enzymes such as SOD2, while simultaneously suppressing SIRT1-mediated activation of PGC-1α and reducing mitochondrial biogenesis (Ferraris et al., 2018). Chronic inflammation upregulates PARP-1 and CD38 expression via NF-κB, both major NAD⁺-consuming enzymes, establishing the vicious cycle of “inflammation → NAD⁺ depletion → mitochondrial decline → further inflammatory amplification” (Katsyuba et al., 2020).

Cytosolic mtDNA released from damaged mitochondria activates the cGAS-STING pathway, driving large-scale SASP transcription (Victorelli et al., 2023). mtROS additionally activates the NLRP3 inflammasome, promoting maturation and secretion of IL-1β and IL-18 (Miwa et al., 2022). Furthermore, the metabolic shift from oxidative phosphorylation to glycolysis in senescent cells further stabilizes the SASP program through mTORC1 activation, representing an important mechanism by which mitochondrial dysfunction is deeply embedded within the chronic inflammation-accelerated aging network (Wiley and Campisi, 2021).

### 5.4 Acceleration of the epigenetic clock (DNA methylation changes)

The “epigenetic clock,” based on DNA methylation patterns, estimates biological age independently of chronological age by tracking age-dependent changes in methylation levels at specific CpG sites, and effectively predicts disease risk and life expectancy (la Torre et al., 2023).

Multiple representative clocks have been developed. The Horvath clock established the first multi-tissue biological age model using 353 CpG sites across diverse tissues; PhenoAge and GrimAge, which incorporate phenotypic parameters, demonstrate superior clinical value in predicting all-cause mortality (Higgins-Chen et al., 2022). The universal mammalian methylation clock, conserved across species, reveals that age-related changes in DNA methylation are intrinsic drivers of aging rather than epiphenomena (Lu et al., 2023). Persistently elevated inflammatory markers are closely associated with accelerated methylation clock rates, with biological age exceeding chronological age (Deryabin and Borodkina, 2023).

Chronic inflammation advances the methylation clock through multiple epigenetic mechanisms. IL-6 activates the JAK/STAT3 pathway, inducing aberrant targeting of DNMT3A/B, causing genome-wide methylation drift: CpG island promoter regions associated with tumor suppressor genes and anti-inflammatory genes undergo hypermethylation, leading to transcriptional silencing of these protective genes; simultaneously, SASP-mediated epigenetic remodeling reduces DNMT1 processivity at replication forks, driving passive methylation loss (Seale et al., 2022).

Persistent NF-κB activation deposits activating histone marks such as H3K27ac at enhancer regions of pro-inflammatory genes through recruitment of CBP/p300 acetyltransferases, establishing open chromatin conformations; concurrently, repressive H3K27me3 marks accumulate at promoter regions of anti-inflammatory and tissue self-renewal genes, establishing an epigenetic asymmetry of “pro-inflammatory gene activation—anti-inflammatory gene silencing” (López-Otín et al., 2023). This asymmetry maintains pro-inflammatory genes in a transcription-ready state after acute inflammation resolves, constituting the molecular basis of chronic inflammation “epigenetic memory.”

SASP-derived IL-6 can activate TET2 demethylase through STAT3 signaling in neighboring cells, driving active demethylation at specific genomic regions; ROS from the SASP directly disrupts normal methylation maintenance mechanisms, synergistically accelerating methylation clock progression at the tissue level (Seale et al., 2022). Thus, the SASP of senescent cells not only propagates senescence at the cellular level through paracrine signaling but also accelerates the biological aging of entire tissues at the epigenomic level.

### 5.5 Immunosenescence and positive feedback with chronic inflammation

Immunosenescence is characterized by low-grade persistent activation of innate immunity and progressive decline in adaptive immune response capacity, forming a mutually reinforcing positive feedback loop with chronic low-grade inflammation, and represents the core immunological mechanism underlying inflammaging formation and maintenance (Santoro et al., 2021).

At the adaptive immune level, thymic involution is the primary driver of immunosenescence. With aging, thymic epithelial cells undergo progressive atrophy, substantially reducing naïve T cell output; persistent antigen exposure and chronic inflammatory signals together drive their accumulation as terminally differentiated effector memory T cells (TEMRA) (Liu et al., 2023). TEMRA cells have almost completely lost proliferative capacity, highly express inhibitory receptors including PD-1, LAG-3, and TIM-3, and display a pronounced exhaustion phenotype; upregulation of p16INK4a in senescent T cells leads to CDK4/6-dependent cell cycle arrest, impairing T cell clonal expansion (Singh et al., 2024). Regulatory T cells (Tregs) suffer dual impairments in function and number under chronic inflammatory conditions. IL-6, through persistent STAT3 activation, can reprogramme Tregs into pro-inflammatory IL-17-secreting cells (Th17-like Tregs), exacerbating the Th17/Treg imbalance (Liu et al., 2023). This mechanism converts cells originally serving anti-inflammatory functions into sources of pro-inflammatory signals, representing a critical distortion node in the inflammaging positive feedback loop.

At the innate immune level, senescent macrophages progressively polarize toward the M1 pro-inflammatory phenotype with lowered TLR4-NF-κB signaling activation thresholds; their efferocytosis capacity declines, resulting in accumulation of apoptotic debris that persistently activates the cGAS-STING pathway (Guo et al., 2022). The NLRP3 inflammasome activation threshold is significantly reduced in senescent immune cells, producing large quantities of IL-1β in response to minimal stimulation, providing an inexhaustible pro-inflammatory driving force for the perpetuation of inflammaging (Fulop et al., 2023).

Chronic inflammation induces premature senescence of immune cells through DNA oxidative damage, accelerated telomere erosion, and elevated ROS; the SASP of senescent immune cells persistently activates NF-κB in surrounding immune cells, achieving self-perpetuating inflammation; simultaneously, impaired Treg function and effector T cell exhaustion cause chronic inflammation to escape normal immune regulation and enter a self-reinforcing state, establishing the irreversible cycle of “chronic inflammation → immune cell senescence → SASP secretion → immune regulatory decline → persistence of chronic inflammation” (Conway and Duggal, 2021).

Gut dysbiosis amplifies systemic inflammatory effects through these mechanisms: LPS persistently activates TLR4-NF-κB signaling in senescent macrophages, deepening M1 polarization; butyrate deficiency further accelerates the Th17/Treg imbalance; metabolic toxins entering the circulation through the leaky gut barrier activate the cGAS-STING pathway, further exacerbating SASP output (Caldarelli et al., 2024). The synergistic “gut dysbiosis-immunosenescence” effect renders inflammaging in older individuals more pronounced and refractory, and represents an important immunological basis for the attenuated responses of elderly populations to infections and vaccines (Santoro et al., 2021).

In summary, chronic inflammation constructs a highly integrated, self-reinforcing aging acceleration network through five intertwined mechanistic pathways: cellular senescence/SASP, telomere damage and genomic instability, mitochondrial dysfunction, epigenetic clock acceleration, and immunosenescence. The SASP provides persistent paracrine inflammatory signals; the DDR-NF-κB axis embeds genomic damage within inflammatory signaling; mitochondrial dysfunction deeply integrates metabolic decline with inflammation through the mtROS-cGAS-STING axis and NAD⁺ depletion; epigenetic reprogramming consolidates and propagates the aging imprint of inflammation at the genomic level; and immunosenescence solidifies chronic inflammation as a pathological state that tissue homeostasis is unable to self-resolve. Targeting multiple key nodes of this integrated network represents the core scientific rationale for developing anti-inflammaging interventional strategies and decelerating the progression of age-related diseases.

## 6 The integrated model of the high-fructose–microbiota–inflammation–aging axis

**Figure 5:**
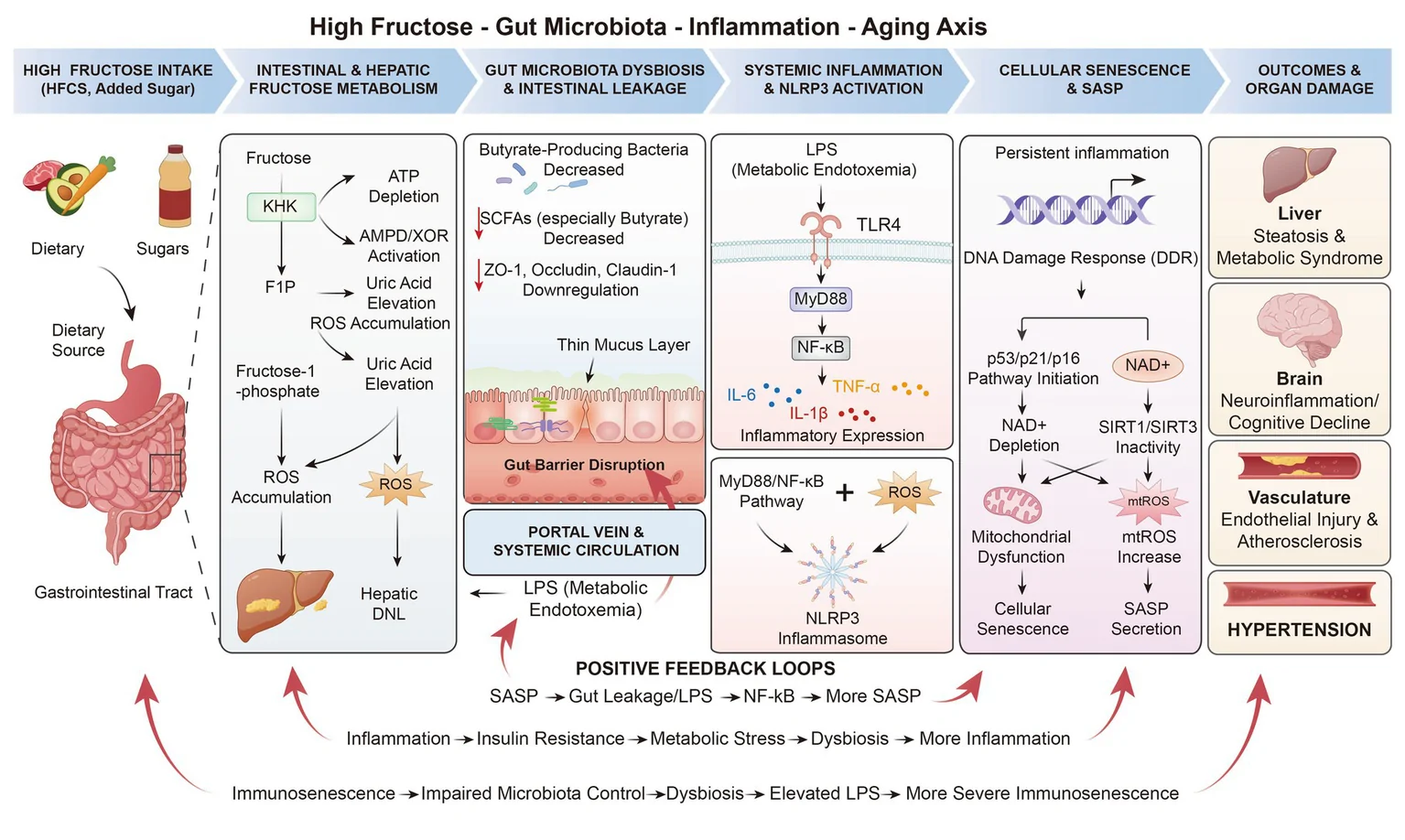
Integrated model of the high-fructose–gut microbiota–inflammation–aging axis driving systemic chronic disease. The pathological cascade proceeds through five sequential, mutually reinforcing stages. Stage 1 (Cytotoxic origin): Excess fructose is rapidly phosphorylated to F1P by ketohexokinase (KHK), depleting ATP and generating uric acid and ROS via AMPD/XOR; F1P metabolites drive hepatic *de novo* lipogenesis, while uric acid upregulates KHK to establish a self-amplifying loop. Stage 2 (Dysbiosis and leaky gut): Selective depletion of butyrate-producing taxa reduces SCFA output; combined with uric acid–driven TLR4/NLRP3–NF-κB activation, this downregulates tight junction proteins and enables LPS translocation into the portal circulation, establishing metabolic endotoxemia. Stage 3 (Systemic inflammation): LPS engages TLR4–MyD88–NF-κB to drive systemic IL-6, TNF-α, and IL-1β overexpression; ROS-mediated NLRP3 inflammasome assembly matures IL-1β/IL-18, sustaining inflammaging across tissues. Stage 4 (Cellular senescence): Persistent inflammation activates DDR and the p53/p21/p16 axis; NF-κB-mediated NAD⁺ depletion via CD38/PARP-1 suppresses SIRT1/SIRT3, exacerbating mitochondrial dysfunction; mtDNA-driven cGAS–STING activation amplifies SASP transcription. Stage 5 (Multi-organ outcomes): The cumulative burden manifests as hepatic steatosis with metabolic syndrome, neuroinflammation with cognitive decline, atherosclerosis, and hypertension. Three positive feedback loops (SASP–leaky gut, inflammation–insulin resistance, immunosenescence–microbiota dysregulation) ensure self-perpetuation of the axis. AMPD, AMP deaminase; cGAS, cyclic GMP-AMP synthase; DDR, DNA damage response; F1P, fructose-1-phosphate; HFCS, high-fructose corn syrup; IL, interleukin; KHK, ketohexokinase; LPS, lipopolysaccharide; mtDNA, mitochondrial DNA; NAD⁺, nicotinamide adenine dinucleotide; NF-κB, nuclear factor κB; NLRP3, NOD-like receptor pyrin domain–containing 3; PARP-1, poly(ADP-ribose) polymerase 1; ROS, reactive oxygen species; SASP, senescence-associated secretory phenotype; SCFAs, short-chain fatty acids; SIRT, sirtuin; STING, stimulator of interferon genes; TLR4, Toll-like receptor 4; TNF-α, tumor necrosis factor α; XOR, xanthine oxidoreductase.

The integrative pathophysiological model linking high-fructose dietary input to accelerated aging through the gut microbiota–inflammation axis is summarized in Figure 5. The cascade proceeds sequentially through five stages—KHK-driven fructose metabolic toxicity, gut microbial dysbiosis with intestinal barrier disruption, LPS–TLR4–NF-κB-driven systemic inflammation with NLRP3 activation, cellular senescence with SASP propagation, and multi-organ pathological outcomes—and is sustained by three self-reinforcing positive feedback loops (SASP–gut leakage, inflammation–insulin resistance, and immunosenescence–microbiota dysregulation), which together translate excess dietary fructose into the shared molecular basis for metabolic syndrome, neurodegeneration, and cardiovascular disease.

### 6.1 The cascade from dietary input to aging phenotype

#### 6.1.1 Stage 1: the cytotoxic origin of fructose metabolism

Unlike glucose, fructose is phosphorylated to fructose-1-phosphate (F1P) by ketohexokinase (KHK), which is highly expressed in the intestine and liver. KHK operates without negative feedback regulation, and under conditions of adequate substrate availability, rapidly depletes intracellular ATP, activates AMP deaminase (AMPD), and generates substantial quantities of uric acid and reactive oxygen species (ROS) via xanthine oxidoreductase (XOR) (Cirillo et al., 2009). Intracellular phosphate depletion suppresses protein synthesis, ROS activates oxidative stress pathways, and uric acid induces cellular inflammation while reducing the expression of intestinal epithelial tight junction proteins through the TLR4/NLRP3–NF-κB signaling axis (Ma et al., 2020). Downstream F1P metabolites drive hepatic *de novo* lipogenesis (DNL) through the acetyl-CoA pathway, while uric acid further induces mitochondrial oxidative stress, inhibits fatty acid β-oxidation, promotes triglyceride accumulation, and ultimately leads to hepatic steatosis (Lanaspa et al., 2012). Uric acid also upregulates KHK expression, establishing a self-amplifying loop of fructose metabolism that activates Kupffer cell-mediated hepatic inflammation, representing an important upstream driver of metabolic endotoxemia (Lanaspa et al., 2012).

#### 6.1.2 Stage 2: gut dysbiosis and barrier disruption

Chronic high-fructose diets profoundly reshape the gut microbial ecosystem. High-fructose syrup (HFS) diets significantly reduce the abundance of butyrate-producing bacteria, including *Faecalibacterium* and *Erysipelatoclostridium*, creating a microbiota profile unfavorable to host metabolic regulation (Beisner et al., 2020). Concurrently, high-fructose feeding results in a global reduction in intestinal short-chain fatty acid (SCFA) levels, with acetate, propionate, and butyrate concentrations all markedly diminished (Tan et al., 2021). Butyrate maintains the normal expression of intestinal epithelial tight junction proteins (ZO-1, Occludin, Claudin-1) through multiple mechanisms including HDAC inhibition; its deficiency directly compromises intestinal barrier integrity and elevates luminal permeability (Peng et al., 2022). Uric acid activates the TLR4/NLRP3–NF-κB signaling axis in intestinal epithelial cells, further suppressing tight junction protein expression and exacerbating intestinal leakage (Ma et al., 2020). The resulting increase in permeability allows substantial translocation of lipopolysaccharide (LPS) from gram-negative bacteria into the portal circulation, inducing metabolic endotoxemia. Evidence demonstrates that chronic high-fat and high-sugar diets can elevate plasma LPS concentrations to 2–3 times baseline levels, sustaining systemic low-grade inflammation (Cani et al., 2007).

#### 6.1.3 Stage 3: LPS-driven systemic inflammation

Circulating LPS binds to the pattern recognition receptor TLR4, activating the MyD88-dependent NF-κB signaling cascade and driving the systemic overexpression of proinflammatory cytokines including TNF-α, IL-6, and IL-1β (Rohr et al., 2020). This low-grade, persistent inflammatory state maintained by metabolic endotoxemia is tightly coupled with the mechanisms of inflammaging: circulating proinflammatory cytokines activate the NLRP3 inflammasome in multiple tissues, sustaining autocrine/paracrine amplification of the senescence-associated secretory phenotype (SASP); chronic NF-κB activation accelerates epigenetic aging (Caldarelli et al., 2024). At the immunological level, sustained LPS stimulation promotes M1 polarization of macrophages and T cell exhaustion, accelerating immunosenescence and further impairing the host’s capacity to regulate dysbiosis (Santoro et al., 2021).

#### 6.1.4 Stage 4: cellular senescence accumulation and aging phenotype

Under persistent inflammatory signals, the ROS burden increases in multiple tissues—including liver, adipose tissue, vascular endothelium, and intestinal epithelium—promoting oxidative telomere damage, DNA damage response (DDR) activation, and induction of the p53/p21/p16 pathway, thereby driving premature cellular senescence. Uric acid and ROS generated through fructose metabolism synergistically promote premature senescence across multiple organ systems at the level of metabolic stress (Johnson et al., 2013). Accumulated senescent cells continuously secrete SASP factors such as IL-6, IL-8, and matrix metalloproteinases (MMPs) into the tissue microenvironment, propagating senescence via paracrine mechanisms and establishing a systemic “senoinflammatory network” (Birch and Gil, 2020). In parallel, KHK-dependent ATP depletion suppresses the activity of NAD⁺-dependent deacetylases (SIRT1/SIRT3), accelerating mitochondrial dysfunction and further amplifying SASP output through the mtROS–cGAS–STING axis (Miwa et al., 2022). The progressive deterioration of aging phenotypes ultimately manifests as multi-tissue functional decline and reduced organ reserve capacity—the core biological basis for heightened susceptibility to chronic disease in older individuals (López-Otín et al., 2023).

In summary, the cascade from “high-fructose dietary input” to “aging phenotype” proceeds sequentially through: KHK metabolic toxicity → uric acid/ROS → gut dysbiosis → SCFA depletion/LPS translocation → TLR4–NF-κB activation → systemic inflammation → NLRP3/DDR → cellular senescence/SASP → tissue functional decline. These stages are not linearly unidirectional; rather, they mutually reinforce one another through multiple positive feedback mechanisms, establishing a pathological state with intrinsic self-sustaining properties.

### 6.2 Mutual reinforcement mechanisms at key nodes

#### 6.2.1 Positive feedback loop 1: SASP → gut dysbiosis

SASP factors released by senescent cells—particularly IL-6 and TNF-α—damage intestinal epithelial tight junctions and directly compromise gut barrier function (Conway and Duggal, 2021). IL-1β inhibits the competitive colonization of beneficial bacteria, exacerbating dysbiosis and reducing SCFA production. The further decline in SCFAs enhances bacterial leakage and amplifies TLR4 activation, sustaining elevated LPS levels and providing a continuous stimulus for NF-κB activation and SASP secretion. This “SASP → barrier leakage → LPS → NF-κB → more SASP” self-amplifying loop constitutes one of the core mechanisms by which inflammation perpetuates itself at the gut–systemic interface (Caldarelli et al., 2024).

#### 6.2.2 Positive feedback loop 2: inflammation → mitochondrial damage → mtROS → more inflammation

IL-6 and TNF-α directly impair mitochondrial electron transport chain complexes I and III, diminishing mitochondrial membrane potential; NF-κB depletes NAD⁺ by upregulating CD38 and PARP-1, thereby attenuating the protective deacetylation of mitochondrial proteins by SIRT3 (Miwa et al., 2022). Mitochondrial dysfunction results in excess mtROS production, activating the cGAS–STING pathway to drive SASP transcription and simultaneously triggering NLRP3 inflammasome activation and release of IL-1β and IL-18, establishing an “inflammation → mtROS → more inflammation” positive feedback spiral. ATP depletion and uric acid accumulation arising from fructose metabolism further amplify this mitochondrial damage (Cirillo et al., 2009).

#### 6.2.3 Positive feedback loop 3: insulin resistance → accumulation of metabolic toxins

IL-6 in the SASP inhibits insulin receptor signaling through JAK/STAT3-mediated IRS-1 serine phosphorylation, while TNF-α via the JNK–IRS-1 pathway cooperatively induces systemic insulin resistance (Liu et al., 2023). Under conditions of insulin resistance, upregulated hepatic DNL exacerbates fructose-induced steatosis; persistent hyperglycemia provides a richer substrate for opportunistic enteric pathogens, further exacerbating dysbiosis and increasing systemic LPS load (Cani et al., 2007). This vicious cycle—“inflammation → insulin resistance → metabolic stress → dysbiosis → more inflammation”—represents one of the core feedback mechanisms by which high-fructose diets drive metabolic syndrome.

#### 6.2.4 Positive feedback loop 4: immunosenescence → impaired microbiota regulation

With advancing immunosenescence, reduced secretory IgA (sIgA) production and impaired regulatory T cell (Treg) function in gut-associated lymphoid tissue (GALT) progressively erode the host’s capacity to regulate microbiota composition (Santoro et al., 2021). Declining microbial diversity perpetuates SCFA deficiency and concurrent degradation of intestinal barrier integrity; persistently elevated LPS and proinflammatory signals further drive immunosenescence, establishing the closed loop: “immunosenescence → impaired microbiota regulation → worsening dysbiosis → elevated LPS → heightened inflammation → further immunosenescence” (Fulop et al., 2023).

### 6.3 Associations with related chronic diseases

#### 6.3.1 Metabolic syndrome

Dysbiosis induced by high-fructose diets markedly reduces SCFA production, impairing insulin signaling in the liver and skeletal muscle and promoting systemic insulin resistance (Beisner et al., 2020). Metabolic endotoxemia activates M1 macrophage infiltration in adipose tissue and liver via TLR4, releasing large amounts of proinflammatory cytokines that further impair pancreatic β-cell function (Cani et al., 2007).

At the level of lipid metabolism, high fructose upregulates key DNL enzymes such as fatty acid synthase (FAS) through ChREBP activation, promoting triglyceride synthesis; fructose-derived uric acid drives hepatic steatosis and visceral fat accumulation by inducing mitochondrial oxidative stress (Lanaspa et al., 2012). Uric acid also activates the renin–angiotensin system and reduces nitric oxide (NO) bioavailability, contributing to fructose-associated hypertension (Staltner et al., 2023). Dietary added sugars selectively deplete immunoregulatory gut microbial taxa, impairing mucosal regulation of intestinal lipid absorption and accelerating the manifestation of the metabolic syndrome phenotype (Kawano et al., 2022).

#### 6.3.2 Neurodegenerative diseases

High-fructose diets reduce the production of SCFAs—particularly butyrate and propionate—that are required to maintain blood–brain barrier (BBB) integrity by inducing gut dysbiosis. SCFAs protect the BBB through mechanisms including HDAC inhibition and upregulation of tight junction protein expression in cerebrovascular endothelial cells (Peng et al., 2022); their depletion increases BBB permeability, facilitating the entry of circulating LPS and proinflammatory cytokines into brain parenchyma.

LPS entering the brain activates microglia, inducing polarization toward the M1 proinflammatory phenotype and triggering robust release of IL-1β and TNF-α, generating neuroinflammatory responses (Zou et al., 2024). Chronic neuroinflammation is one of the central driving mechanisms of neurodegenerative diseases, including Alzheimer’s disease (AD). *Faecalibacterium prausnitzii* abundance correlates positively with cognitive assessment scores (MoCA-J) and is significantly reduced in individuals with mild cognitive impairment (MCI) compared with healthy controls; administration of isolated *F. prausnitzii* strains to AD mouse models ameliorates cognitive impairment, suggesting potential therapeutic value for gut–brain axis interventions (Ueda et al., 2021).

The gut microbiota-derived metabolite trimethylamine N-oxide (TMAO) also exhibits neurotoxic properties: TMAO increases BBB permeability and activates NF-κB and NLRP3 signaling in the hippocampus, and elevated TMAO levels are associated with progressive cognitive decline (Zhen et al., 2023).

#### 6.3.3 Cardiovascular disease

The microbiota-dependent TMAO metabolic pathway represents a central molecular mechanism underlying the cardiovascular impact of the high-fructose–microbiota–inflammation axis. Gut microbiota metabolize dietary choline and carnitine to trimethylamine (TMA), which is transported via the portal circulation to the liver, where it is oxidized to TMAO by flavin monooxygenase 3 (FMO3). Elevated TMAO promotes atherosclerosis through multiple mechanisms including impaired reverse cholesterol transport (RCT), enhanced macrophage foam cell formation, activation of vascular endothelial inflammation, and increased platelet reactivity (Canyelles et al., 2023). Epidemiological evidence demonstrates a significant positive association between high circulating TMAO levels and the risk of major adverse cardiovascular events (MACE) (Oktaviono et al., 2023).

Uric acid generated from fructose metabolism directly injures the vascular endothelium by reducing eNOS activity, diminishing NO bioavailability, and increasing endothelial ROS production through NADPH oxidase activation (Staltner et al., 2023). Systemic low-grade inflammation driven by gut dysbiosis—characterized by elevated LPS, IL-6, and TNF-α—promotes the formation and destabilization of atherosclerotic plaques by upregulating vascular endothelial adhesion molecules (VCAM-1, ICAM-1) (Rohr et al., 2020).

NAD⁺ depletion and mitochondrial dysfunction induced by high-fructose diets manifest in cardiomyocytes as aberrant metabolic reprogramming toward glycolysis, representing an important metabolic basis for heart failure progression (Miwa et al., 2022). Hyperuricemia cooperates with fructose-induced metabolic dysregulation to drive hypertension through activation of the renin–angiotensin–aldosterone system (RAAS), further compounding the cardiovascular risk burden (Staltner et al., 2023).

In conclusion, the high-fructose–microbiota–inflammation–aging axis, with gut dysbiosis and systemic inflammation as its central hubs, translates specific dietary risk factors into a shared molecular pathological basis for metabolic syndrome, neurodegenerative disease, and cardiovascular disease through multiple cascade pathways and positive feedback loops. This axis provides an integrative theoretical framework for dietary intervention strategies aimed at decelerating the progression of aging-related diseases (López-Otín et al., 2023).

## 7 Potential intervention strategies

**Figure 6:**
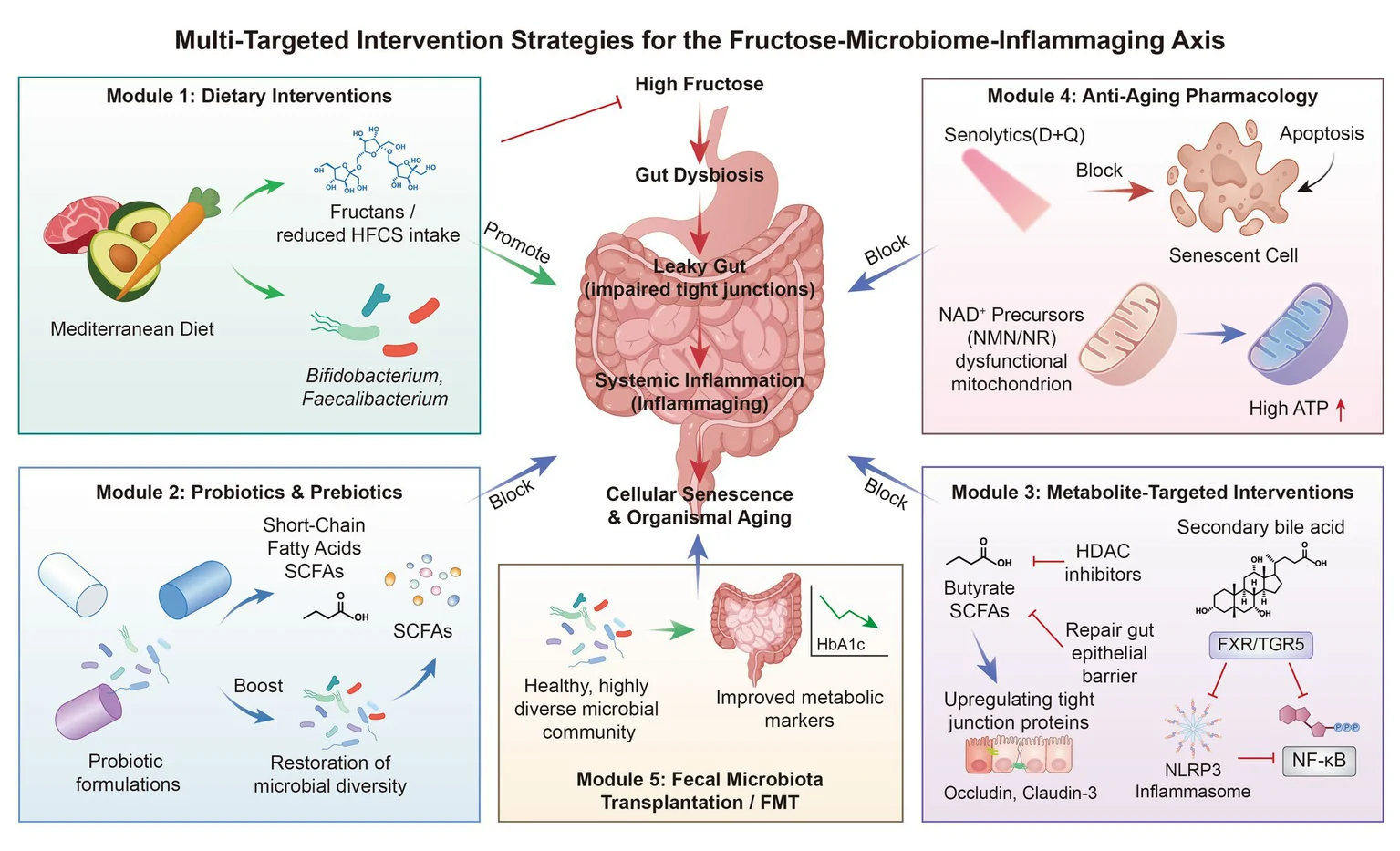
Multi-targeted intervention strategies along the high-fructose–gut microbiota–inflammaging axis. Five complementary intervention modules act on distinct nodes of the pathological cascade (gut dysbiosis → leaky gut → systemic inflammation → cellular senescence). Module 1—Dietary interventions (upstream input control): Restriction of HFCS/added sugars combined with the Mediterranean diet reduces metabolic toxicity and promotes expansion of *Bifidobacterium* and *Faecalibacterium prausnitzii*, strengthening barrier and microbial foundations. Module 2—Probiotics and prebiotics (ecological reshaping): Probiotic strains and prebiotic substrates (inulin, oligofructose) restore microbial diversity and endogenously elevate SCFA output. Module 3—Metabolite-targeted interventions (downstream signaling repair): Exogenous butyrate upregulates tight junction proteins via HDAC inhibition, while restored secondary bile acid–FXR/TGR5 signaling suppresses NLRP3 and NF-κB activation. Module 4—Anti-aging pharmacology (terminal node clearance): Senolytics (dasatinib + quercetin) eliminate p16INK4A/p21CIP1-positive senescent cells, reducing SASP burden; NAD⁺ precursors (NMN, NR) restore mitochondrial function via SIRT1/SIRT3 reactivation. Module 5—FMT (systems-level reconstruction): Transfer of a healthy, diverse donor microbiota fundamentally reconstructs intestinal ecology, with documented short-term improvements in HbA1c and insulin sensitivity. Together, these modules form a complementary “upstream-blocking, midstream-restoring, downstream-clearing” framework, with multi-target individualized regimens guided by baseline microbiota and inflammatory biomarker stratification representing the central direction for clinical translation. D + Q, dasatinib plus quercetin; FMT, fecal microbiota transplantation; FXR, farnesoid X receptor; HbA1c, glycated hemoglobin; HDAC, histone deacetylase; HFCS, high-fructose corn syrup; NAD⁺, nicotinamide adenine dinucleotide; NF-κB, nuclear factor κB; NLRP3, NOD-like receptor pyrin domain–containing 3; NMN, nicotinamide mononucleotide; NR, nicotinamide riboside; SASP, senescence-associated secretory phenotype; SCFAs, short-chain fatty acids; TGR5, Takeda G protein-coupled receptor 5.

The five complementary intervention strategies targeting distinct nodes of the high-fructose–gut microbiota–inflammaging axis are summarized in Figure 6. Dietary modification reduces upstream pathogenic input; probiotic and prebiotic interventions reshape intestinal microbial ecology; bile acid and SCFA metabolite-targeting repairs downstream barrier and inflammatory dysfunction; pharmacological agents clear senescent cells and replenish key metabolic pathways; and FMT achieves systemic microbiota reconstruction—together forming an integrated “upstream-blocking, midstream-restoring, downstream-clearing” therapeutic framework.

### 7.1 Dietary approaches: restricting fructose intake and alternative strategies

Excess dietary fructose represents a key upstream driver of the gut dysbiosis–inflammation–aging cascade, making dietary intervention the most direct strategy for targeting the origin of this pathological axis. Restricting intake of added sugars—particularly high-fructose corn syrup (HFCS)—can reduce ketohexokinase (KHK)-mediated metabolic injury, attenuate uric acid and reactive oxygen species (ROS) overproduction, and alleviate hepatic *de novo* lipogenesis (DNL) burden. A double-blind, crossover, inpatient metabolic-unit randomized controlled trial (RCT) in subjects with obesity demonstrated that 14 days of excess fructose consumption (approximately 20% of total caloric intake) produced no significant changes in fecal microbiota composition, intestinal permeability, or indices of endotoxemia, suggesting that chronic cumulative exposure is more pathogenically relevant than short-term high-dose intake (Alemán et al., 2021).

The Mediterranean dietary pattern (MedDiet) represents one of the best-evidenced alternative dietary strategies. Characterized by high consumption of plant-based foods, monounsaturated fatty acids from olive oil, and polyphenols, published evidence indicates indicates that MedDiet adherence is positively correlated with increases in gut microbial diversity and short-chain fatty acid (SCFA) production, and promotes the growth of beneficial taxa including *Bifidobacterium*, *Faecalibacterium prausnitzii*, and *Roseburia*, thereby reinforcing gut barrier integrity (Kimble et al., 2023). Multiple studies have confirmed that adherence to the MedDiet is associated with enrichment of *Faecalibacterium* and *Prevotella* genera, reductions in inflammatory markers, and improvements in cardiometabolic health outcomes (Khavandegar et al., 2024). In older populations, the MedDiet modulates the abundance of SCFA-producing bacteria, exerts protective effects against frailty progression and age-related gut dysbiosis, and its efficacy is partly determined by baseline microbiota composition (Ticinesi et al., 2024).

High-fiber diets constitute an important complementary strategy. Dietary fiber provides preferential fermentable substrate for butyrate-producing bacteria, thereby endogenously increasing intestinal butyrate levels and enhancing the gut microbiota’s capacity for SCFA production (Baxter et al., 2019). Fasting and intermittent caloric restriction enrich anti-inflammatory gut commensals, particularly *Faecalibacterium*, improve insulin signaling, and exert multifaceted regulatory effects on the gut microbiota–inflammation axis, as demonstrated in human intervention studies (Forslund, 2023).

### 7.2 Modulation by probiotics and prebiotics

Probiotic and prebiotic interventions target the gut microbiota–inflammation–aging axis at a second therapeutic level by directly reshaping intestinal microbial ecology and modulating SCFA production capacity.

Supplementation with probiotics—predominantly *Bifidobacterium* and *Lactobacillus* species—increases the abundance of beneficial bacteria in the elderly gut. Synbiotic formulations (probiotics combined with prebiotics) may further augment SCFA production by providing dedicated fermentable substrates for SCFA-producing taxa, and exert anti-inflammatory effects on inflammaging-related markers. Both probiotic and prebiotic interventions display strain-specific modulatory effects on gut microbial composition, and exhibit potential for attenuating the age-related dysbiosis that underlies chronic low-grade inflammation (Sánchez et al., 2022). Emerging evidence indicates that the gut microbiota influences frailty syndrome in older adults through its effects on SCFA-mediated signaling in the central nervous system, muscle, and bone; and that probiotic and prebiotic interventions targeting microbial composition may potentially alleviate frailty-related symptoms (Wang et al., 2024).

At the clinical level, a multicenter, double-blind, placebo-controlled RCT (*n* = 200) demonstrated that a prebiotic blend of inulin and oligofructose significantly improved frailty scores, gait speed, and handgrip strength, accompanied by increased fecal *Bifidobacterium* abundance and alterations in microbial metabolite profiles, directly linking microbiota changes to functional improvements in the frailty phenotype (Yang J. et al., 2024).

Age-associated changes in gut microbiota composition—characterized by a progressive decline in beneficial taxa such as *Faecalibacterium prausnitzii* and an expansion of pro-inflammatory species—are closely associated with the development of frailty (Lim and Nam, 2023). This pattern underscores the importance of appropriate timing and treatment duration in probiotic and prebiotic interventions aimed at achieving sustained microbial ecological modulation, and highlights the necessity of tailoring strategies to individual baseline microbiota profiles.

### 7.3 Metabolite-targeted interventions: bile acid modulation and SCFA supplementation

Metabolite-targeted interventions focus on two core pathways: bile acid (BA) signal modulation and exogenous SCFA supplementation, both of which constitute critical downstream effector nodes of the high-fructose–gut microbiota–inflammation axis.

### 7.4 Bile acid signal modulation

The gut microbiota converts primary bile acids into secondary bile acids—including deoxycholic acid (DCA) and lithocholic acid (LCA)—through bile salt hydrolase (BSH) activity and 7α-dehydroxylation. Secondary BAs function as endocrine signaling molecules by activating the farnesoid X receptor (FXR) and the Takeda G protein-coupled receptor 5 (TGR5). TGR5 activation stimulates glucagon-like peptide-1 (GLP-1) secretion from intestinal L cells, thereby improving insulin sensitivity; FXR activation suppresses NOD-like receptor thermal protein domain-associated protein 3 (NLRP3) inflammasome assembly and nuclear factor κB (NF-κB) signaling, exerting anti-inflammatory and metabolic protective effects. High-fructose diet-induced gut dysbiosis reduces secondary BA production, impairing these protective signaling pathways and exacerbating both intestinal inflammation and metabolic dysfunction (Zhao et al., 2023). FXR/TGR5 axis activation is directly associated with intestinal barrier integrity and inflammation suppression, and targeting this axis has shown therapeutic potential in models of inflammatory bowel disease and metabolic disorders (Han et al., 2023). The bidirectional interplay between bile acids and gut microbiota is increasingly recognized as a promising target for precision intervention in metabolic syndrome (Zhu et al., 2024).

### 7.5 Exogenous SCFA supplementation

Butyrate is the best-characterized SCFA therapeutic target. The protective mechanisms of exogenous butyrate include: acting as a histone deacetylase (HDAC) inhibitor to regulate intestinal epithelial gene expression and promote synthesis of tight junction proteins including claudin-3 and occludin, thereby restoring the intestinal barrier; activating GPR41/GPR43 receptors to produce anti-inflammatory effects. In aged animal models, progressive depletion of butyrate-producing bacteria leads to aberrant free fatty acid receptor 3 (FFAR3) signaling, and butyrate supplementation reverses aged microbiota transplantation-induced gut inflammation, increased intestinal permeability, and cognitive decline (Mishra et al., 2024). A randomized controlled study demonstrated that oral sodium butyrate combined with inulin supplementation improved gut microbiota composition and intestinal barrier function in obese subjects, evidenced by increased proportions of *Akkermansia* and *Bifidobacterium* (Roshanravan et al., 2018). Clinical evidence supports a clear therapeutic role for butyrate in suppressing intestinal mucosal inflammation and repairing the gut barrier; the development of microencapsulated sodium butyrate (MSB) formulations has further improved stability and targeted delivery efficiency (Hodgkinson et al., 2023).

Combining SCFA supplementation with prebiotics confers synergistic effects: prebiotics endogenously elevate SCFA levels by promoting the proliferation of butyrate-producing bacteria, while exogenous supplementation compensates for SCFA deficiency under conditions of severe dysbiosis, potentially enabling more sustained inhibition of the inflammatory cascade through combined use.

### 7.6 Prospects for combined anti-inflammatory and anti-aging pharmacotherapy

Pharmacological interventions targeting the high-fructose–gut microbiota–inflammation–aging axis are primarily focused on four therapeutic strategies: elimination of senescent cells (senolytics), suppression of the senescence-associated secretory phenotype (senomorphics), NAD⁺ metabolic replenishment, and mTOR/AMPK pathway modulation.

### 7.7 Senolytic therapy

The combination of dasatinib and quercetin (D + Q) represents the most clinically advanced senolytic intervention. In a Phase 1 open-label clinical trial in patients with diabetic kidney disease, a 3-day oral course of D + Q reduced the burden of p16INK4A- and p21CIP1-expressing senescent cells in adipose tissue within 11 days, accompanied by decreases in circulating SASP factors including IL-1α, IL-6, MMP-9, and MMP-12, providing the first direct in-human demonstration of senescent cell clearance (Hickson et al., 2019). In aged mouse models, D + Q selectively eliminated senescent cells from perigonadal white adipose tissue (pgWAT), suppressed expression of SASP-associated pro-inflammatory genes (mcp1, tnf-α, il-1β, il-6, cxcl2, and cxcl10), and significantly improved fasting blood glucose, glucose tolerance, and systemic lipid metabolism, suggesting therapeutic potential for age-related metabolic syndrome (Islam et al., 2023).

### 7.8 NAD⁺ precursors and metabolic regulatory drugs

Nicotinamide mononucleotide (NMN) and nicotinamide riboside (NR), as NAD⁺ precursors, restore mitochondrial function through activation of SIRT1/SIRT3 deacetylases, inhibit NLRP3 inflammasome activation, and reduce SASP-associated cytokine output. Metformin activates AMP-activated protein kinase (AMPK), inhibits mitochondrial complex I, and modulates gut microbiota composition, collectively attenuating chronic inflammation and delaying multi-organ aging. The mTORC1 inhibitor rapamycin promotes autophagy-mediated clearance of damaged cellular components and delays the emergence of cellular senescence phenotypes. A comprehensive review of human clinical trials covering metformin, NAD⁺ precursors, GLP-1 receptor agonists, TORC1 inhibitors, spermidine, senolytics, probiotics, and anti-inflammatory agents evaluated available clinical evidence for these candidate anti-aging interventions, providing an important framework for optimizing multi-target combination strategies (Guarente et al., 2024).

These agents carry dose-dependent safety challenges in systemic use, including the potential antagonism of mTOR inhibition on muscle protein synthesis and wound healing, mild attenuation of aerobic exercise benefits by metformin, and competitive utilization of orally administered NMN by gut microbiota; these complex factors must be comprehensively weighed in the design of individualized precision therapeutic regimens.

### 7.9 The therapeutic potential of fecal microbiota transplantation

Fecal microbiota transplantation (FMT) transfers the entire intestinal microbial community from a healthy donor to the recipient’s gut, fundamentally reconstructing the microbial ecological structure, and thus provides a systems-level intervention pathway for the high-fructose–gut microbiota–inflammation–aging axis.

### 7.10 Clinical evidence in metabolic syndrome

A meta-analysis including nine RCTs (*n* = 303 participants) demonstrated that FMT produced significant short-term metabolic benefits (≤6 weeks): fasting blood glucose was reduced (MD = −0.12 mmol/L, 95% CI: −0.23, −0.01), HbA1c decreased (MD = −0.37 mmol/mol, 95% CI: −0.73, −0.01), insulin levels fell significantly, and HDL cholesterol increased (MD = 0.07 mmol/L, 95% CI: 0.02, 0.11). FMT was well tolerated without serious adverse events; however, significant weight reduction was not observed, suggesting that FMT primarily improves glycemic regulation and insulin sensitivity rather than exerting a direct anti-obesity effect (Qiu et al., 2023).

A secondary analysis of a Phase 2 randomized, double-blind, placebo-controlled clinical trial in adults with severe obesity and metabolic syndrome demonstrated that the metabolic response to FMT was strongly determined by baseline recipient microbiota composition. Insulin resistance responders (improved HOMA-IR) exhibited significantly higher engraftment rates of donor-specific microbes—including *Faecalibacterium intestinalis*, *Roseburia* spp., and *Christensenellaceae* spp.—and had significantly lower baseline *Prevotella* abundance than non-responders (*p* = 0.02), supporting the use of recipient baseline microbiota profiling as a predictive indicator for precision donor-recipient matching (Zhang et al., 2024).

### 7.11 Limitations and future directions

FMT application continues to face multiple challenges: high inter-individual variability in therapeutic outcomes due to donor-recipient microbiota compatibility; difficulty maintaining short-term metabolic benefits over the long term; differential effects of administration route (colonoscopy, nasojejunal tube, oral capsule) on microbial engraftment efficiency; and the need for standardization of donor screening criteria and regulatory frameworks. Comprehensive evaluation indicates that FMT holds modest promise as an adjunctive therapy for metabolic syndrome, but the limited sample sizes and evidence quality of existing trials necessitate larger, longer-follow-up, high-quality RCTs before definitive conclusions can be drawn (Horvath et al., 2024).

Taken together, the five categories of intervention strategies outlined above operate across distinct levels of the high-fructose–gut microbiota–inflammation–aging axis: dietary modification reduces upstream pathogenic input; probiotic and prebiotic interventions reshape intestinal microbial ecology; bile acid and SCFA metabolite-targeting repairs downstream barrier and inflammatory dysfunction; pharmacological agents address senescent cells and key metabolic pathways; and FMT achieves systemic microbiota reconstruction. A critical direction for future research is the development of multi-target individualized combination regimens based on precision phenotypic stratification incorporating baseline microbiota profiling, metabolomics, and inflammatory biomarker characterization (Guarente et al., 2024).

## 8 Conclusions and perspectives

### 8.1 Summary of major findings

This review has examined the multilevel cascade linking high-fructose diet (HFD), gut microbiota dysbiosis, chronic inflammation, and aging, establishing an integrated conceptual framework of the high-fructose–gut microbiota–inflammation–aging axis and providing a comprehensive synthesis of its molecular mechanisms, self-reinforcing feedback loops, and intervention strategies. At the level of metabolic toxicity initiation, excess fructose intake drives massive ATP depletion through ketohexokinase (KHK)-mediated phosphorylation; the resulting overproduction of uric acid and reactive oxygen species (ROS) subsequently activates the NLRP3 inflammasome and NF-κB signaling, promoting hepatic *de novo* lipogenesis (DNL) and steatosis. Concurrently, chronic fructose loading progressively depletes butyrate-producing bacteria (*Faecalibacterium prausnitzii*, *Roseburia* spp., and related taxa), reduces intestinal short-chain fatty acid (SCFA) output, downregulates tight junction proteins (claudin-3 and occludin), and compromises gut barrier integrity, culminating in lipopolysaccharide (LPS) translocation into the systemic circulation and the establishment of metabolic endotoxemia (Jung et al., 2022). At the level of inflammatory amplification, LPS activates the TLR4–MyD88–NF-κB axis, sustaining elevated systemic pro-inflammatory cytokines (TNF-α, IL-6, and IL-1β) and driving chronic low-grade inflammation (inflammaging), which is further amplified through bidirectional crosstalk with the senescence-associated secretory phenotype (SASP): SASP factors damage the intestinal epithelium, exacerbating dysbiosis, while dysbiosis in turn reinforces SASP output by reducing SCFA production and increasing LPS flux, thus establishing a self-perpetuating feed-forward loop. These pathological processes are deeply intertwined with multiple hallmarks of aging—including mitochondrial dysfunction, accumulating cellular senescence, immune dysregulation, and dysbiosis—all of which have been incorporated into the most recent expanded hallmarks of aging framework, conceptually establishing the axis described in this review as a central mechanistic node of aging biology (López-Otín et al., 2023). At the level of disease association, the high-fructose–gut microbiota–inflammation–aging axis converges with multiple aging-related disease spectra through distinct downstream effectors: metabolic syndrome [insulin resistance, non-alcoholic fatty liver disease (NAFLD), and hyperuricemia], neurodegenerative disorders (Aβ/tau pathology and cognitive decline), and cardiovascular disease [elevated trimethylamine N-oxide (TMAO) and atherosclerosis]. The bidirectional promotion between intestinal inflammaging and gut barrier dysfunction has been confirmed across multiple species as a conserved hallmark of aging, further supporting the gut as the central anatomical and functional node of this axis (Zhang et al., 2023). At the interventional level, this review has catalogued five categories of strategies targeting distinct nodes of the axis: dietary modification [fructose restriction, Mediterranean dietary pattern (MedDiet), high-fiber diet, and intermittent fasting], probiotics and prebiotics (restoration of microbial ecology), metabolite-targeted interventions (bile acid FXR/TGR5 signaling and SCFA supplementation), anti-inflammatory and anti-aging pharmacotherapy (D + Q senolytics, NAD⁺ precursors, metformin, and rapamycin), and fecal microbiota transplantation (FMT; systemic microbiota reconstitution). Available clinical evidence indicates that these approaches can produce measurable benefits in glycemic regulation, insulin sensitivity, SASP attenuation, and microbiota diversity enhancement, although the magnitude of response for each strategy is substantially modulated by individual baseline microbiota composition (Guarente et al., 2024).

### 8.2 Limitations of current research

Despite significant advances in mechanistic understanding of the high-fructose–gut microbiota–inflammation–aging axis, the existing evidence base carries important limitations that constrain the external validity of its conclusions and the clinical translatability of derived intervention strategies. Regarding causal inference, the majority of mechanistic evidence originates from animal models, predominantly rodents, and faces substantial barriers to human extrapolation: notable species differences exist in KHK isoform expression profiles, rates of intestinal fructose absorption and hepatic metabolism, and baseline gut microbiota composition. A 14-day high-fructose randomized controlled trial (RCT) in individuals with obesity found no significant changes in gut microbiota composition or indices of endotoxemia, indicating that the effects of short-term fructose exposure in humans are considerably weaker than animal model predictions, and that the pathogenic relevance of chronic long-term exposure remains to be directly established through high-quality human longitudinal studies (Alemán et al., 2021). Regarding methodological limitations, current microbiota research relies predominantly on 16S rRNA amplicon sequencing, which provides limited resolution of microbial function; inter-study comparability is further compromised by differences in sequencing platforms, variable region selection, and bioinformatic pipelines. Integrated multi-omics approaches combining metagenomics, metatranscriptomics, and metabolomics remain underutilized, precluding adequate dissection of the independent contributions of compositional versus functional microbiota changes to the inflammatory phenotype. Notably, aging has been shown to enhance the immunogenic signaling potential of gut microbiota—characterized by elevated activation capacity of pathogen-associated molecular patterns (PAMPs) such as LPS and flagellin—thereby amplifying low-grade inflammation; however, the causal directionality of the relationship between dysbiosis and inflammaging (i.e., whether microbial alterations are the primary driver or a downstream consequence of aging) remains to be resolved through well-designed longitudinal studies (Caetano-Silva et al., 2024). Regarding biomarker standardization, key indicators of inflammaging and cellular senescence—including SASP factor panels, p16INK4A, p21CIP1, and intestinal permeability markers—lack internationally validated standardized assay protocols, limiting comparability across clinical studies; the integration of epigenetic aging clocks with gut microbiota-based metrics remains at an early exploratory stage and has yet to yield a scalable clinical assessment framework. Regarding the quality of intervention evidence, existing clinical trials are broadly limited by small sample sizes, short follow-up durations, and high heterogeneity in intervention protocols. The meta-analysis of FMT for metabolic syndrome encompassed only nine RCTs with 303 participants and predominantly short-term follow-up, leaving the durability of metabolic benefits unestablished; human trials of D + Q senolytics remain at Phase 1, and large-scale efficacy data are lacking; and the strain-specificity and inter-individual variability of probiotic and prebiotic effects preclude generalization of current clinical evidence into unified treatment recommendations (Qiu et al., 2023). Finally, available studies suffer from limited population diversity, with most cohorts drawn from Western or specific East Asian populations, providing inadequate representation across age strata, dietary backgrounds, comorbidity profiles, and racial or genetic diversity; the profound inter-individual variability in gut microbiota composition—shaped by genetics, dietary history, medication use, and lifestyle factors—further constrains the translation of population-level intervention evidence to individual clinical applications.

### 8.3 Future research directions

Addressing the limitations outlined above will require concerted advances across several complementary levels. In terms of basic research infrastructure and cohort development, there is an urgent need for rigorously designed prospective longitudinal cohorts that integrate serial metagenomics, metabolomics, and inflammatory biomarker profiling under controlled dietary conditions, enabling direct causal-temporal characterization of the relationships between chronic fructose exposure, gut dysbiosis, and aging phenotypes; such studies should enroll multi-age, multi-dietary, and multi-center heterogeneous populations to yield findings with genuine external validity. In terms of technological advancement, as metagenomics, metatranscriptomics, metabolomics, and proteomics continue to mature, integrated multi-omics analyses will enable functional-level dissection of microbiota contributions to the high-fructose–inflammation–aging axis, moving beyond purely taxonomic descriptions; artificial intelligence (AI) and machine learning algorithms hold substantial promise for integrating multidimensional omics data, identifying key microbial biomarkers, and predicting individual responses to specific interventions—including FMT, probiotics, and dietary modification—thereby advancing the precision medicine application of the gut microbiome (Rozera et al., 2025). In terms of biomarker integration, future studies should combine measures of senescent cell burden (p16INK4A and p21CIP1), epigenetic aging clocks, and SASP factor profiles with gut microbiota functional metrics (SCFA concentrations, circulating LPS levels, and microbiota diversity indices), constructing a composite assessment framework capable of simultaneously reflecting microbiota status and biological age to accurately define the optimal timing and targets for axis-level intervention; given that intestinal barrier dysfunction has been established as an evolutionarily conserved hallmark of aging, the development and validation of non-invasive assessment tools for gut permeability should be prioritized (Salazar et al., 2023). In terms of clinical validation of intervention strategies, given that individual responses to dietary modification, probiotic/prebiotic supplementation, senolytics, and FMT are all substantially determined by baseline microbiota characteristics, future clinical trials should adopt a “microbiota-stratified randomization” design, prospectively stratifying participants according to baseline microbiota composition, metabolomic profiles, and inflammatory phenotypes to match optimal intervention combinations; the synergistic efficacy and safety of multi-target combinatorial strategies—such as dietary intervention combined with synbiotics and senolytics—require evaluation within rigorous trial frameworks, with particular attention to high-risk populations including elderly individuals with metabolic syndrome and frailty syndrome (Zhang et al., 2024). In terms of translational medicine and public health, as senolytic clinical trials expand into further indications (including Phase 2/3 studies in Alzheimer’s disease and osteoarthritis) and as human evidence accumulates for candidate agents including GLP-1 receptor agonists and mTOR inhibitors, the high-fructose–gut microbiota–inflammation–aging axis is poised to emerge as an actionable precision intervention target in the near future (Guarente et al., 2024). At the public health level, restricting dietary added sugars and high-fructose corn syrup (HFCS) in ultra-processed foods remains the most cost-effective primary prevention strategy; integrating this approach with microbiome-targeted health promotion may decelerate the incidence of aging-related chronic diseases at the population level.

In summary, the integrative characterization of the high-fructose–gut microbiota–inflammation–aging axis not only elucidates a critical mechanistic link between contemporary dietary patterns and accelerated aging, but also provides a robust theoretical foundation for multilevel, precision-guided aging intervention strategies; as multi-omics technologies, AI-assisted analytical platforms, and rigorously designed clinical trials continue to converge, the field is well-positioned to achieve substantive breakthroughs within the framework of precision geromedicine, ultimately advancing the paradigm shift from “delaying disease” toward “promoting healthy aging” (Caldarelli et al., 2024).
